# Abnormal Interactions between Perifollicular Mast Cells and CD8+ T-Cells May Contribute to the Pathogenesis of Alopecia Areata

**DOI:** 10.1371/journal.pone.0094260

**Published:** 2014-05-15

**Authors:** Marta Bertolini, Federica Zilio, Alfredo Rossi, Patrick Kleditzsch, Vladimir E. Emelianov, Amos Gilhar, Aviad Keren, Katja C. Meyer, Eddy Wang, Wolfgang Funk, Kevin McElwee, Ralf Paus

**Affiliations:** 1 Department of Dermatology, University of Lübeck, Lübeck, Germany; 2 Department of Dermatology, University of Münster, Münster, Germany; 3 Department of Internal Medicine and Medical Specialties, University “La Sapienza”, Rome, Italy; 4 Department of Gynaecology and Obstetrics, University of Rostock, Rostock, Germany; 5 Department of Pharmacology, Clinical Pharmacology and Biochemistry, Chuvash State University Medical School, Cheboksary, Russia; 6 Laboratory for Skin Research, Rappaport Faculty of Medicine, Technion–Israel Institute of Technology, Haifa, Israel; 7 Flieman Medical Center, Haifa, Israel; 8 Department of Dermatology and Skin Science, University of British Columbia, Vancouver, British Columbia, Canada; 9 Klinik Dr. Koslowski, Munich, Germany; 10 Institute for Inflammation and Repair, University of Manchester, Manchester, United Kingdom; University of Maryland School of Medicine, United States of America

## Abstract

Alopecia areata (AA) is a CD8+ T-cell dependent autoimmune disease of the hair follicle (HF) in which the collapse of HF immune privilege (IP) plays a key role. Mast cells (MCs) are crucial immunomodulatory cells implicated in the regulation of T cell-dependent immunity, IP, and hair growth. Therefore, we explored the role of MCs in AA pathogenesis, focusing on MC interactions with CD8+ T-cells *in vivo,* in both human and mouse skin with AA lesions. Quantitative (immuno-)histomorphometry revealed that the number, degranulation and proliferation of perifollicular MCs are significantly increased in human AA lesions compared to healthy or non-lesional control skin, most prominently in subacute AA. In AA patients, perifollicular MCs showed decreased TGFβ1 and IL-10 but increased tryptase immunoreactivity, suggesting that MCs switch from an immuno-inhibitory to a pro-inflammatory phenotype. This concept was supported by a decreased number of IL-10+ and PD-L1+ MCs, while OX40L+, CD30L+, 4–1BBL+ or ICAM-1+ MCs were increased in AA. Lesional AA-HFs also displayed significantly more peri- and intrafollicular- CD8+ T-cells as well as more physical MC/CD8+ T-cell contacts than healthy or non-lesional human control skin. During the interaction with CD8+ T-cells, AA MCs prominently expressed MHC class I and OX40L, and sometimes 4–1BBL or ICAM-1, suggesting that MC may present autoantigens to CD8+ T-cells and/or co-stimulatory signals. Abnormal MC numbers, activities, and interactions with CD8+ T-cells were also seen in the grafted C3H/HeJ mouse model of AA and in a new humanized mouse model for AA. These phenomenological *in vivo* data suggest the novel AA pathobiology concept that perifollicular MCs are skewed towards pro-inflammatory activities that facilitate cross-talk with CD8+ T-cells in this disease, thus contributing to triggering HF-IP collapse in AA. If confirmed, MCs and their CD8+ T-cell interactions could become a promising new therapeutic target in the future management of AA.

## Introduction

Alopecia areata (AA), one of the most common human autoimmune disorders, represents a T-cell-dependent organ-specific autoimmune disease that is clinically characterized by sudden, mostly focal, hair loss [1], [2]. The immunopathogenesis of AA and the relevant hair follicle (HF) autoantigen(s) remain to be clarified. However, transfer of CD8(+) cells alone induces localized AA-like hair loss in the C3H/HeJ mouse model [1], [3], while CD8+ T-cell depletion abrogates AA onset in a rat model [4]. AA can be also induced by IL-2 stimulated NKG2D+/CD56+ immunocytes, many of which are CD8+, in human skin [5].

Growing (anagen) HFs exhibit relative immune privilege (IP) based on the suppression of MHC class I molecules and the over-expression of IP guardians like TGFβ1/2 [1], [2], [6]–[9]. The development of AA requires that the normal IP of growing HFs collapses, induced by excessive release of interferon-γ (IFNγ) for example [5], [10], [11] (for prevalent AA pathogenesis concepts, see [2]).

The perifollicular inflammatory cell infiltrate in lesional AA HFs contains lymphocytes (CD8+ and CD4+ T-cells), natural killer cells, some Langerhans cells and increased numbers of mature, histochemically detectable mast cells (MC) [12]–[18]. While T-cells, particularly CD8+ lymphocytes, have long been a focus of AA research (e.g. [3]–[5], [14], [19]–[24], MCs have received much less attention (Background S1 in File S1).

While MCs have long been viewed as primary effector cells of innate immunity, more recent research has revealed that they also play a key role in connecting innate and adaptive immune responses [25]–[34]. In fact, MCs can even control antigen-specific CD8+ T-cell responses, namely in murine experimental autoimmune encephalitis (EAE) [35], another organ-specific autoimmune disease characterized by IP collapse. Consequently, the pathobiological contribution of MCs to autoimmune disorders such as type 1 diabetes and multiple sclerosis is attracting increasing attention [25], [26], [31], [36]–[39].

This recent development made it compelling to further examine the enigmatic role of MCs in AA, whose number has been reported to be increased in lesional human AA skin by some authors [12], [14]–[16]. Such a focus on MCs in AA was further encouraged by the fact that MCs are recognized hair growth modulators [40]–[44], and that the HF mesenchyme in humans and mice harbours resident MC progenitor cells, from which fully functional, mature skin MCs can differentiate *in loco* [45]–[47].

MCs are now appreciated to exert a dual immunoregulatory role [25]–[31], [34], [38], [48]: Under physiological circumstances, MCs may be primarily immuno-inhibitory, thus contributing to the maintenance of IP and peripheral tolerance [27], [31], [34], [37], [48]–[53] and therefore, possibly, to the maintenance of HF-IP [2], [53]. However, as MCs are primed to rapidly secrete proinflammatory ‘danger’ signals, their role can quickly convert into a tolerance-breaking, potentially autoimmunity-promoting one, such as during allograft rejection and EAE [25], [26], [31], [38], [39], [48], [52]–[54].

Given the recognized key role of CD8+ T-cells in AA pathogenesis [1]–[4], [20], one main research challenge, therefore, is to characterize MC-CD8+ T-cell interactions in human AA pathobiology. However, for human MCs, one is currently restricted to phenomenological studies (see Discussion). In order to keep such analyses as instructive and clinically relevant as possible, we have combined the *in situ-*analysis of AA lesions in AA patients with the analysis of grafted C3H/HeJ AA mice [55], [56] and of experimentally induced AA lesions in previously healthy human skin transplanted onto SCID mice [5], [57]. In these independent, mutually complementary AA models, we have addressed the specific questions summarized in **Table 1**, using quantitative (immuno-)histomorphometry and triple-immunostaining techniques, as well as a range of relevant MC markers (Background S2 in File S1), so as to gauge skin MC function *in situ*.

**Table 1:** Experimental design and specific questions addressed.

Model	Question addressed	Investigated read-out parameters
**Lesional and non-lesional skin from AA patients versus healthy skin**	Do perifollicular MCs and their activities increase in AA?	Evaluation of MC number using c-Kit, TB and Ki-67/tryptase stainings. Evaluation of MC degranulation using TB and Ki-67/tryptase stainings. Evaluation of MC proliferation using Ki-67/tryptase, c-Kit/tryptase and Ki-67/c-Kit stainings.
	Do MCs switch to a pro-inflammatory phenotype in AA?	Evaluation of TGFβ1 and tryptase contents within MCs using TGFβ1/c-Kit and Ki-67/tryptase stainings. Evaluation of MC number positive for OX40L, CD30L, 4-1BBL, ICAM-1, IL-10 or PD-L1 using corresponding triple-staining.
	Do MCs interact with CD8+ T-cells in AA?	Evaluation of MC number in close contact with CD8+ T-cells using CD8/tryptase and CD8/c-Kit.
	Are the observed interactions between MCs and CD8+ T-cells likely to be pro-inflammatory or immuno-inhibitory?	Evaluation of MC number either degranulating or positive for OX40L, CD30L, 4-1BBL, ICAM-1, IL-10 or PD-L1 when in close contact with CD8+ T-cells using corresponding triple-staining.
**Grafted C3H/HeJ mice ** [55], [56]	Do perifollicular MCs and their activities increase in AA affected mice?	Evaluation of MC number using c-Kit/CD8 and mMCP6/CD8, stainings. Evaluation of MC degranulation using mMCP6/CD8 staining.
	Are MCs and MC-CD8+ T-cell interactions also abnormal in the C3H/HeJ AA mouse model?	Evaluation of MC number in close contact with CD8+ T-cells using mMCP6/CD8 and c-Kit/CD8 stainings.
**Humanized-mouse model of AA ** [5], [57]	Can key findings made in the skin of AA patients with respect to excessive MC number/activities and MC-CD8+ T-cell interactions be reproduced in experimentally induced AA-like lesions in previously healthy human skin?	Evaluation of MC number using c-Kit and tryptase/CD8, stainings. Evaluation of MC number degranulation using tryptase/CD8 staining. Evaluation of MC number in close contact with CD8+ T-cells using tryptase/CD8 staining.

## Material and Methods

### Human specimens

Human lesional and non-lesional scalp skin biopsies were obtained from 7 AA patients (n = 7, lesional skin, n = 4, non-lesional skin) after written patients' consent, and internal review board (Department of Internal Medicine, n. 11, 29-01-13) and ethic committee (n. 2973, 28-11-13) approvals, University “La Sapienza” of Rome.

An additional 23 human lesional scalp skin biopsies from AA patients were obtained from archival paraffin blocks (up to 10 years old) from biopsies that had been taken exclusively for diagnostic purposes from the Dermatopathology Paraffin Block Collection, Dept. of Dermatology University of Luebeck, after ethics committee approval (University of Luebeck, n. 13-007, 13-03-13). It was not possible to obtain the written consent as most patients were not traceable after such a long period. Consequently, anonymized use of these tissue blocks without formal written consent was approved by the ethics committee.

Clinically “healthy” frontotemporal human skin scalp samples obtained from 23 women without a record of AA (mean age: 55 years) undergoing cosmetic facelift surgery were used as controls after ethics committee approval (University of Luebeck, n. 06-109, 18-07-06) and written patient consent.

As positive control tissues for different immunostaining protocols, anonymized human tonsil and placenta tissue samples were obtained from the Dept. of Pathology, University of Luebeck, with ethics approval (n. 06-109, 20-01-2009), without the necessity of written patient consent.

All experiments were performed according to Helsinki guidelines.

### Mice

Grafted C3H/HeJ AA model: 13 weeks-old female C3H/HeJ mice were purchased from The Jackson Laboratory, Bar Harbor, Maine USA and housed in the British Columbia University facility. The mice were transplanted with healthy hairy or alopecic skin isolated from older C3H/HeJ donors as previously described [55], [56]. Most of the mice transplanted with alopecic lesions developed AA, here called AA mice (mAA), while a few mice failed to develop AA, here called failed-grafted mice (fAA). Mice transplanted with normal hairy skin did not develop AA, here called sham-grafted mice (mSH). For comparison non-transplanted mice were also used, here called normal mice (NM). After about one year, the mice were killed and the skin samples were collected from the mid to lower back close to the midline, avoiding the skin graft area, fixed in cryo-matrix and frozen in liquid nitrogen (thus, “mAA” skin refers to new areas of alopecia arising apart from the engraftment site on the younger graft recipient mice). This experiment was performed following ethics approval by the University of British Columbia Animal Care Committee (n. A10-0166, 16-08-13). The skin samples were then shipped to the University of Lübeck for (immuno-)histomorphometry analysis.

Humanized AA mouse model: Paraffin sections of human skin were obtained from 6 C.B-17/IcrHsd-scid-bg mice (derived from 2 independent experiments) that had been transplanted with healthy human scalp skin and subsequently injected intradermally with allogeneic, IL-2 or PHA-treated, PBMCs from healthy donors enriched for NKG2D+/CD56+ cells (for details, see [5], [57]. Mice that received an injection of IL-2-treated NKG2D+/CD56+ cell-enriched PBMCs clinically and histologically developed characteristic AA lesions in the transplanted, previously healthy hair-bearing human skin, while control mice receiving NKG2D+/CD56+-enriched cells cultured with PHA instead of IL-2 failed to develop AA in the human skin transplants. Human skin samples were obtained after informed patient consent and ethics approval (n. 919970072, 13-05-97) from the Flieman Medical Center and the Ministry of Health, Israel, and the study was performed in accordance with the Declaration of Helsinki Principles. Animal care and research protocols were in accordance with institutional guidelines and were approved by the Technion Institute Committee on Animal Use (n. IL-087-08-2011, 08-11).

### Immunohistology

For detection of single antigens (for details see **Table 2**, Material and methods S3 and Table S1 in File S1), the skin sections were immunostained following established protocols [58], [59] by using the avidin-biotin complex method and the corresponding chromogen. Similar protocols were used for each protein stained in double or triple-immunohistochemistry (IHC) in which we serially stained the sections for each protein (for details see **Table** **2**, Material and methods S3 and Table S1 in File S1). To perform double- [60] and triple-immunofluorescence (IF) labelling, we used the appropriate primary antibody (Table S1 in File S1) and secondary antibodies conjugated to the correct fluorophore (**Table 2**). Each immunostaining protocol was also conducted with the appropriate positive and negative controls (Figure S1 in File S1), which confirmed both the sensitivity and specificity of the immunoreactivity (IR) patterns reported here.

**Table 2:** Immunostainings.

Antigen(s)	Specimen	Antigen retrieval/fixation	1^st^ detection system	2^nd^ detection system	3^rd^ detection system	Counter staining
**C-Kit**	Human	Sodium Citrate	ABC-HRP, DAB			Hematoxylin
**Ki-67/tryptase**	Human	Sodium Citrate	ABC-HRP, DAB	ABC-AP, SIGMAFAST		
**C-Kit/tryptase**	Human	Sodium Citrate	IF, FITC,	IF, Rho		DAPI
**Ki-67/c-Kit**	Human	Sodium Citrate	ABC-HRP, DAB	ABC-AP, SIGMAFAST		Hematoxylin
**Ki-67/tryptase**	Human	Sodium Citrate	IF, Rho	IF, FITC		DAPI
**CD8/tryptase**	Human, Hu-mouse	Sodium Citrate	ABC-HRP, DAB	ABC-AP, SIGMAFAST		Hematoxylin
**TGFβ1/c-Kit**	Human	Sodium Citrate	IF, Rho or IF, FITC	IF, FITC or IF, Rho		DAPI
**TGFβ1**	Human	Sodium Citrate	ABC-HRP, DAB			
**OX40L/CD8/tryptase**	Human	TRIS-EDTA	ABC-HRP, AEC	ABC-AP, Vector Blue	ABC-AP, SIGMAFAST	
**CD30L/c-Kit/CD8**	Human	Sodium Citrate	ABC-HRP, AEC	ABC-AP, Vector Blue	ABC-HRP, DAB	
**4–1BBL/c-Kit/CD8**	Human	Sodium Citrate	ABC-HRP, AEC	ABC-AP, Vector Blue	ABC-HRP, DAB	
**ICAM-1/CD8/tryptase**	Human	Sodium Citrate	ABC-HRP, AEC	ABC-AP, Vector Blue	ABC-AP, SIGMAFAST	
**IL-10/c-Kit/CD8**	Human	TRIS-EDTA	ABC-HRP, AEC	ABC-AP, Vector Blue	ABC-HRP, DAB	Methyl-green
**PD-L1/c-Kit/CD8**	Human	Sodium Citrate	ABC-HRP, AEC	ABC-AP, Vector Blue	ABC-HRP, DAB	
**CD200/CD8/tryptase**	Human	TRIS-EDTA	DAB	ABC-AP, Vector Blue	ABC-AP, SIGMAFAST	
**C-kit**	Hu-mouse	Sodium Citrate	ABC-AP, SIGMAFAST			Hematoxylin
**C-Kit/CD8**	Mouse	Acetone	ABC-HRP, AEC	ABC-AP, SIGMAFAST		Hematoxylin
**mMCP6/CD8**	Mouse	Acetone	Envision-HRP, AEC	ABC-HRP, DAB		Hematoxylin

List of all immunostainings which were performed and relevant details (For list of primary antibodies, see Table S1 in File S1).

ABC-AP: Avidin-biotin complex, alkaline phosphatase; ABC-HRP: Avidin-biotin complex, horseradish peroxidase; AEC: 3-amino-9-ethylcarbazole; DAB: 3,3′-diaminobenzidine, DAPI: diamidino-2-phenylindole, Envision-HRP: Envision- horseradish peroxidise; FITC: Fluorescein isothiocyanate; IF: immunofluorescence, SIGMAFAST™: Fast Red TR/Naphthol AS-MX tablets, Rho: Rhodamine.

### Histochemistry

Mature MCs were also visualized histochemically with 1% toluidine blue (TB) as described [47], [58].

### Quantitative (immuno-)histomorphometry

**Figure 1:**
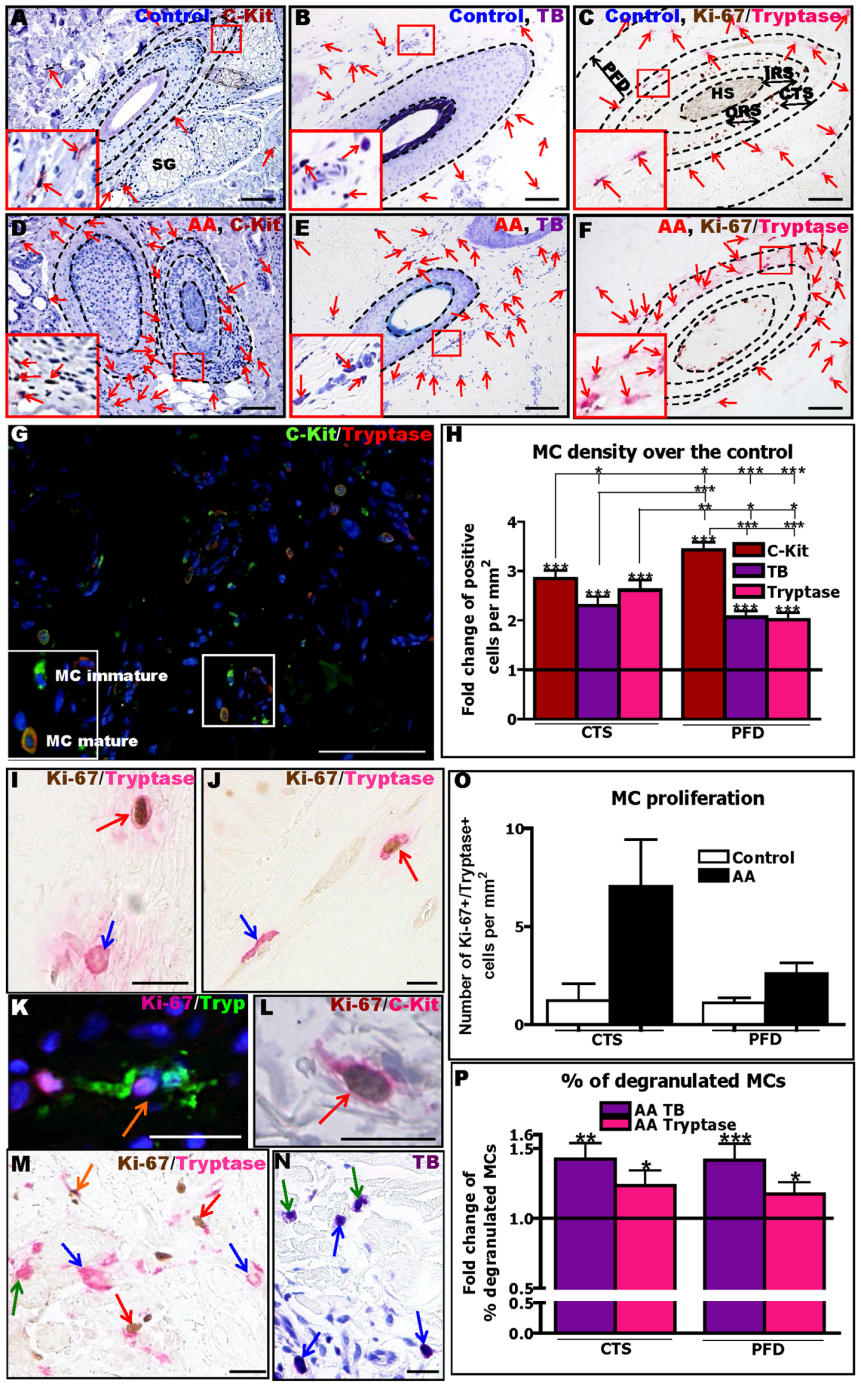
Human AA lesions show increased density, proliferation and degranulation of perifollicular MCs. The immunohistochemical identification and evaluation of MCs by c-Kit (A,D), TB (B,E) or Ki-67/tryptase (C,F) revealed a strong increase of MC numbers in AA (D–F) compared to control healthy (A–C) skin. Red arrows indicate MCs. C-Kit/tryptase double-IF shows immature c-Kit+ MCs (stained in green) and mature c-Kit+/tryptase+ MCs in AA skin (stained in green and red) (G). See inserted panels in the bottom left of each Figure for higher magnification views of the area highlighted in the small boxes. Reference area for the quantitative analysis using (immuno-)histomorphometry for cell counting in the connective tissue sheath (CTS) and perifollicular dermis (PFD). CTS+PFD represents the total area including the space demarcated up to 200 µm from the HF basement membrane (C,F). Fold change of MC density detected by c-Kit, TB and tryptase stainings (H). Black line indicates the control. Analysis derived from 69–81areas (HFs) of 11–17 AA patients and from 50–69 areas (HFs) of 5–7 healthy controls, ±SEM, *p≤0.05, **p≤0.01, ***p≤0.001, Mann-Whitney-U-Test or Student t-test (for c-Kit, TB and tryptase compared to respective controls and for comparing bars between CTS and PFD), Kruskal-Wallis test (p<0.0001) followed by Dunn's test (for comparing c-Kit, TB and tryptase within CTS and PFD). Identification of MCs by Ki-67/tryptase IHC (I,J,M), Ki-67/tryptase IF (K), Ki-67/c-Kit IHC (L) and TB (N) showing non-degranulating, non proliferating MCs (blue arrows), degranulating, non-proliferating MCs (green arrows), non-degranulating MCs undergoing proliferation (red arrows) and proliferating degranulating MCs (orange arrows). Quantitative analysis of MC proliferation by Ki-67/tryptase IHC (O). Analysis derived from 81 areas (HFs) of 17 AA patients and 50 areas (HFs) of 7 healthy controls, ±SEM, Mann-Whitney-U-Test (ns). Quantitative analysis of MC degranulation by TB histochemistry and Ki-67/tryptase IHC (P). Black line indicates the control. Analysis derived from 69–81 areas (HFs) of 11–17 AA patients and 50–69 areas (HFs) of 5–7 healthy controls, ±SEM, *p≤0.05, **p≤0.05, ***p≤0.001 Mann-Whitney-U-Test (compare to control), Mann-Whitney test (TB compare to tryptase) (ns). Scale bars: 100 µm (A–G) and 20 µm (I–N) Connective tissue sheath (CTS), hair shaft (HS), inner root sheath (IRS), outer root sheath (ORS), perifollicular dermis (PFD), toluidine blue (TB), sebaceous gland (SG).

The cell densities of MCs, CD8+ T-cells and MC-CD8+ T-cell interactions were evaluated in the HF connective tissue sheath (CTS) and the peri-follicular dermis (PFD) by (immuno-)histomorphometry. The total reference area (CTS+PFD) included all tissue within a distance of up to 200 µm from the HF basement membrane in a human skin section (**Figure 1C,F**). In murine skin, positive cells were counted in a perifollicular tissue area within 50 µm of the HF [58], [61]. MCs with more than five granules located outside of the cell membrane were regarded as “degranulated” [43], [47], [58]. The staining intensity of TGFβ1 and tryptase of individual MCs was evaluated by quantitative analysis [58]–[59], using NIH image J software (National Institute of Health, Bethesda, Maryland).

### Statistical analysis

All data were analyzed by Student's *t-* or Mann-Whitney-U- test when only two groups were compared, or by One Way-ANOVA or Kruskal-Wallis test followed by Bonferroni's or Dunn's multiple comparison tests, respectively, when more than two groups were analyzed, using GraphPad (GraphPad Prism version 4.00 for Windows; GraphPad Software, San Diego, CA, USA). Data are expressed as mean ±SEM; p values of <0.05 were regarded as significant.

## Results

### Human AA lesions show increased density, proliferation and degranulation of perifollicular MCs

First, we sought to resolve the controversy in the published literature on whether or not the number of MCs is increased in lesional AA skin [12], [14]–[16], [62]. Quantitative analysis of MC numbers in human AA skin by TB histochemistry and c-Kit and Ki-67/tryptase IHC, unequivocally revealed a significant increase in MC density in the HF mesenchyme (CTS) and in the surrounding perifollicular dermis (PFD) compared to both healthy control skin (**Figure** **1A–F, H**) and non-lesional AA skin (Figure S2A in File S1). The variable absolute MC numbers, dependent on the MC detection method used (**Figure 1A–H**) (Resul S4 in File S1), are in line with the r**e**cognized presence of distinct MC subpopulations in human skin [47], .

Next, we asked whether the increased MC number resulted from enhanced MC proliferation. Although quantitative Ki-67/tryptase double-IHC (**Figure 1I,J,M**) revealed a slightly higher number of proliferating mature MCs in both CTS and PFD in AA skin compared to healthy control skin, this did not reach significance (**Figure 1O**). Double-IHC for Ki-67+/c-Kit+ cells (**Figure 1L**) suggested a trend towards slightly increased MC proliferation in AA skin (Figure S2B in File S1). This raised the possibility that the substantial numeric increase of MCs in lesional AA skin not only results from increased intracutaneous proliferation of MCs, but also from increased proliferation and maturation of resident MC progenitor cells in human skin [45]–[47], and/or from an enhanced influx of MC progenitors from the circulation.

Quantitative analysis of TB histochemistry and tryptase IHC (**Figure 1I–K,M,N**) showed significantly more MC defined as “degranulated” in the CTS and PFD of AA skin than in healthy control skin (**Figure 1P**). Thus, AA lesions are associated with a greatly enhanced activation status of skin MCs *in situ*.

In order to assess whether MC numbers and granulation status are AA phase-dependent, AA patients were divided into three groups, based on their histological features [65] (“acute”, “subacute” and “chronic” AA) and on clinical evaluation criteria supplied by the attending dermatologist, using all three MC detection methods (c-Kit, TB, tryptase). This demonstrated a maximal increase of MC density in lesional PFD compared to healthy controls for the subacute AA group (Figure S3A-E in File S1 and data not shown), though significance was only reached when the number of c-Kit+ cells was analysed (Figure S3E in File S1).

### MCs in AA skin are skewed towards a pro-inflammatory phenotype

**Figure 2:**
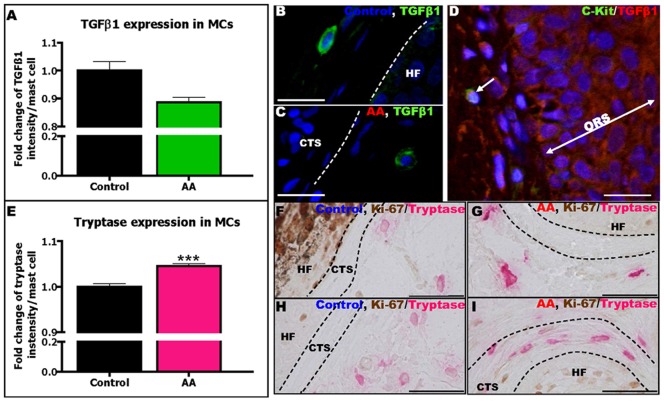
AA MCs contain less TGFβ1 and more tryptase compared to control MCs. Quantitative analysis of TGFβ1 IR in perifollicular MCs in AA patients compared to controls (A). Analysis derived from 272 MCs around 29 HFs of 10 AA patients and 175 MCs around 19 HFs of 2 healthy controls, ±SEM, Mann-Whitney-U-Test (ns). Representative pictures of TGFβ1+ MCs in human scalp skin of controls (B) and AA patients (C) stained by TGFβ1(green)/c-Kit(red) double-staining. Representative picture of TGFβ1(red)/c-Kit(green) double-staining (D). Quantitative analysis of tryptase IR in perifollicular MCs in AA patients compared to controls (E). Analysis derived from 272 MCs around 41 HFs of 14 AA patients and 182 MCs around 19 HFs of 2 healthy controls, ***p≤0.001, ±SEM, Mann-Whitney-U-Test. Representative pictures of tryptase+ MCs in human scalp skin of control (F,H) and AA patients (G,I) stained by Ki-67/tryptase double-staining. Scale bars: 20 µm (B–D) and 50 µm (F–I). Connective tissue sheath (CTS), hair follicle (HF), outer root sheath (ORS).

Subsequently, we searched for phenotypic indications for changes in MC function *in situ*. MCs can release potent immunoinhibitors such as TGFβ1 [29], [30], [34], [51], [66]–[68], which is also one of the most important guardians of HF-IP [1], [6]–[9], [69]. Interestingly, TGFβ1 IR was lowered in the outer root sheath (ORS) of lesional AA-HFs (Result S5 and Figure S4A–C in File S1), consistent with compromised HF-IP [1], [2], [59], [69]. Therefore, we examined whether the TGFβ1 expression in perifollicular MCs was also abnormal. Indeed, TGFβ1/c-Kit double-IF revealed that perifollicular MCs in lesional AA skin showed reduced TGFβ1 content compared to controls (**Figure 2A–D**). This suggests that the TGFβ-based immuno-inhibitory phenotype of perilesional MCs is attenuated in AA.

[LOOSESR]Tryptase is a pro-inflammatory, trypsin-like protease stored together with heparin within MCs and released upon degranulation [29], [30], [47], [48], [70]–[74]. Tryptase functions are mostly mediated by signalling via the PAR-2 receptor [70], [71], [73]–[75] and by activating other proteases such as collagenases [31], [72]. Additional analyses showed that the MC content of tryptase was significantly up-regulated in perifollicular MCs in lesional AA skin compared to controls (**Figure 2E–I**). This further supports the concept that perifollicular MCs in AA switch from an immuno-inhibitory to a pro-inflammatory phenotype at some stage during AA pathogenesis.

### The number of CD8+ T-cells that are in close contact with MCs is significantly increased in AA

**Figure 3:**
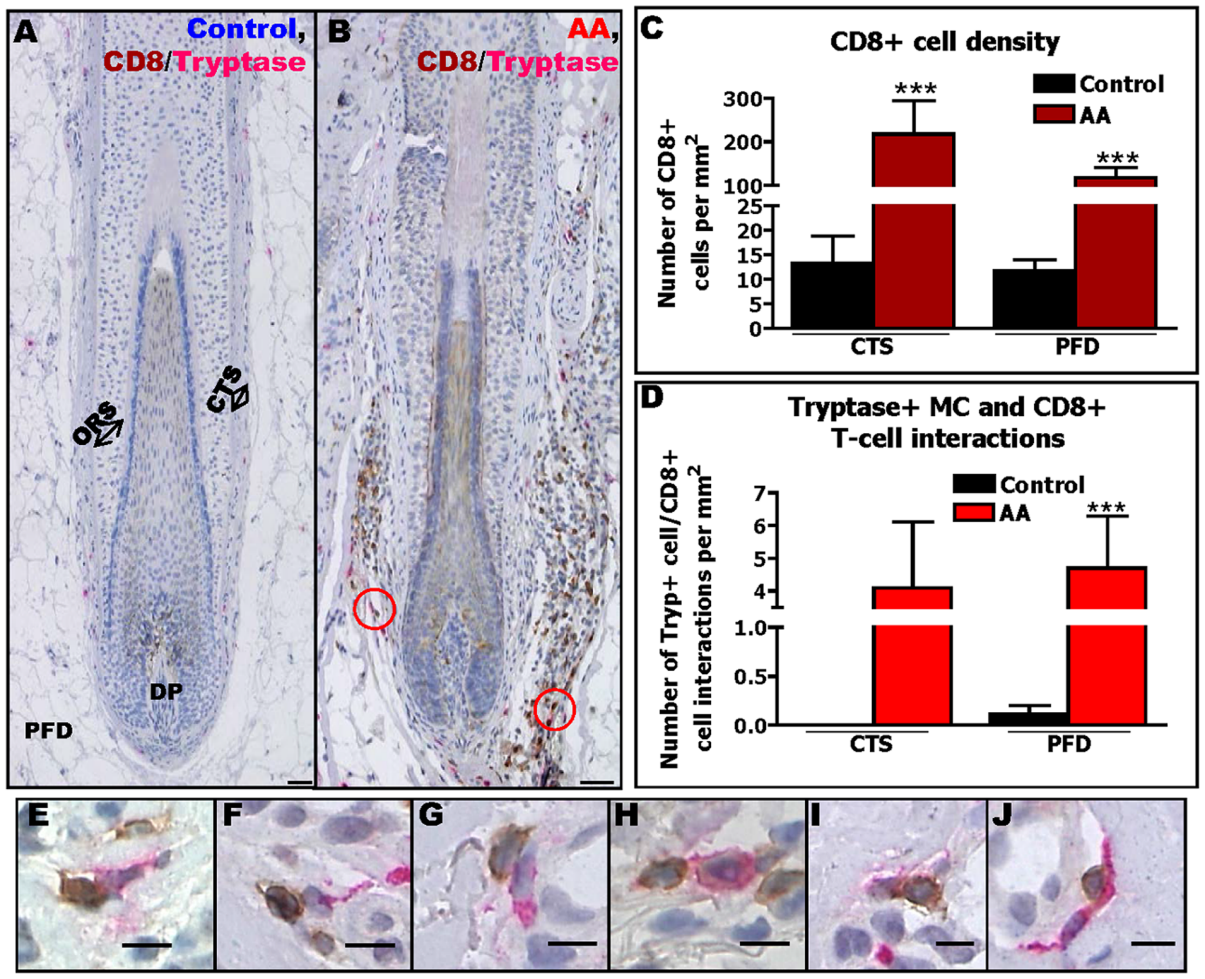
The number of perifollicular CD8+ T-cells and MC-CD8+ T-cell interactions are increased in AA. Immunohistochemical identification of tryptase+ MCs and CD8+ T-cells in human scalp skin of controls (A) and AA patients (B). Quantitative analysis of CD8+ T-cells (C) and of their interactions with tryptase+ MCs (D). Analysis derived from 56 areas (HFs) from 13 AA patients and 44 areas (HFs) of 7 healthy controls, ***p≤0.001, ±SEM, p value was calculated by Mann-Whitney-U-Test. Non-degranulating MCs (E,G,H) and degranulating MCs (F,I,J) close to CD8+ T-cells Scale bars: 50 µm (A–B) and 10 µm (E–J). Connective tissue sheath (CTS), dermal papilla (DP), outer root sheath (ORS), perifollicular dermis (PFD).

In view of the accepted key role of CD8+ T-cells in AA pathogenesis [1]–[4], [20] and the fact that MCs can activate CD8+ T-cells [25], [26], [29], [30], [35], [76], [77], we subsequently analyzed MC-CD8+ T-cell contacts in human AA. As expected from the literature [14], [19], [21], [24], [78], [79], the number of CD8+ T-cells in the perifollicular mesenchyme was significantly higher in lesional compared to non-lesional AA (data not shown) and to healthy anagen HFs (Figure 3A–C).

Subsequent analyses provided the first evidence that MCs co-localize with CD8+ T-cells around the HF in AA skin significantly more frequently (**Figure 3B,D–J**) than in healthy control skin (**Figure 3A,D**) and non-lesional AA skin (data not shown). Moreover, during these interactions, almost 50% of MCs were found to be degranulated, as assessed by tryptase IHC (**Figure 3F,I,J**). These perifollicular MCs were strongly MHC class I-positive (Result S6 and Figure S5A–D in File S1) indicating their capacity to present autoantigens to cognate CD8+ T-cells.

### MCs in lesional AA skin up-regulate co-stimulatory molecules for CD8+ T-cells

This led to the question how MCs and CD8+ T-cells may interact in AA. Since MCs can express many cell surface molecules that are either co-stimulatory or inhibitory for CD8+ T-cells (see e.g., [25], [26], [28]–[31], [34], [51]), MC-CD8+ T-cell interactions can be regulated and fine-tuned by multiple different signalling partners. As a first attempt towards dissecting these interactions *in situ*, we established triple-IHC for MCs, CD8+ T-cells and some of the best-characterized co-stimulatory molecules known to modulate MC-CD8+ T-cells interactions [25], [26], [28]–[31], [76], [77].

**Figure 4:**
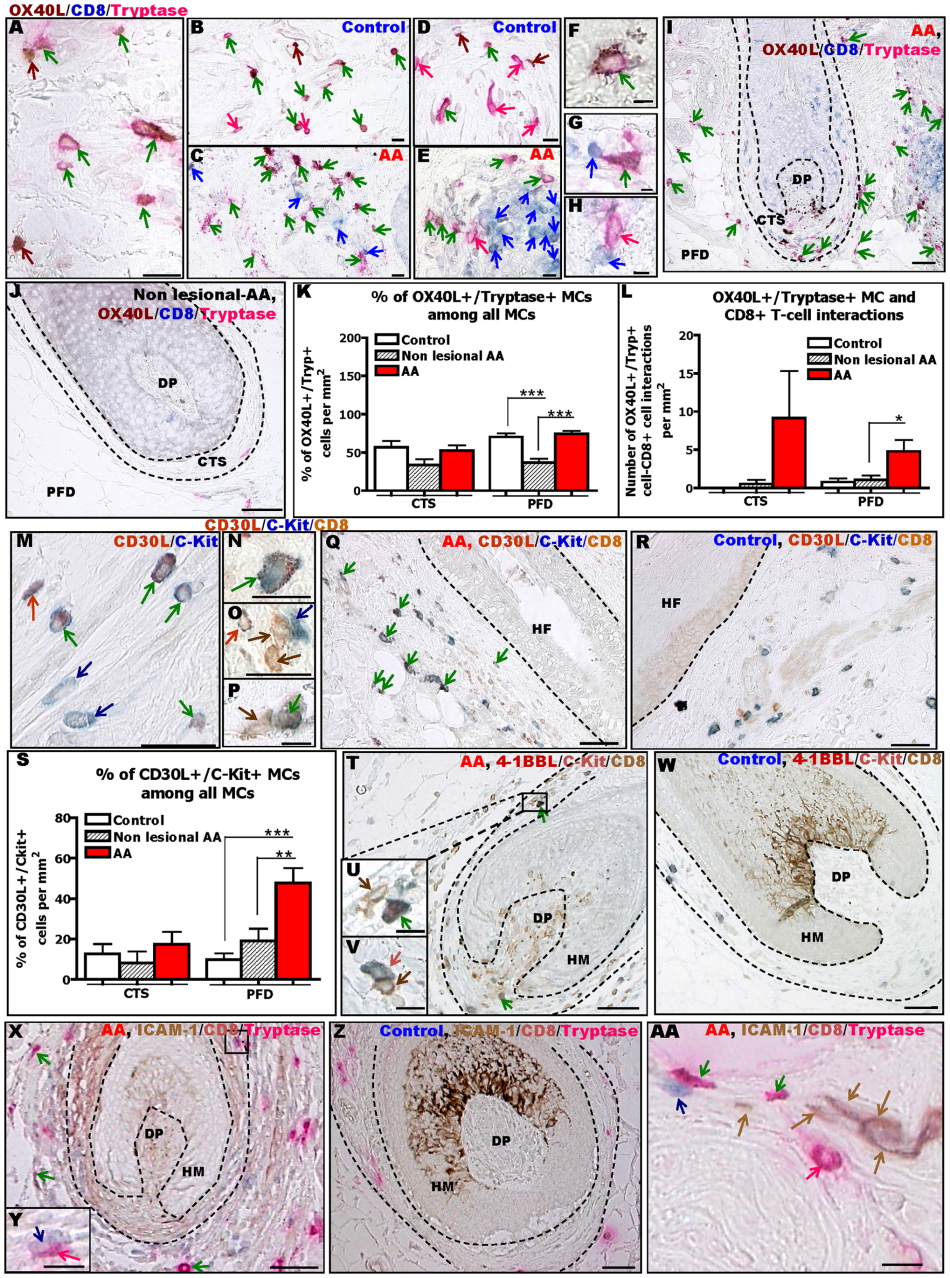
MCs in lesional AA skin up-regulate co-stimulatory molecules for CD8+ T-cells. Immunohistochemical identification of OX40L+/tryptase+ MCs, detected using OX40L/CD8/tryptase staining (A–E) and CD30L+/c-Kit+ MCs, detected using CD30L/c-Kit (M), showing the expression pattern of OX40L (A–E) and CD30L (M) within MCs, in human scalp skin of controls (A,B,D,M) and AA patients (C,E). Higher magnification of OX40L+/tryptase+ (F) and CD30L+/c-Kit+ (N) MCs. Representative pictures of OX40L+ (G) and OX40L− (H) (detected by tryptase) and CD30L− (O) and CD30L+ (P) (detected by c-Kit) MCs interacting with CD8+ T-cells. Immunohistochemical identification of OX40L+/tryptase+ (I,J) and CD30L+/c-Kit+ (Q,R) MCs and CD8+ T-cells in human HFs from lesional (I,Q), non-lesional (J) AA and healthy (R) skin. Brown cells are OX40L+ (A–J) or CD8+ (M–R) cells (brown arrows), blue cells are CD8+ (A–J) or c-Kit+ (M–R) cells (blue arrows), pink cells are tryptase+ cells (A–J) (pink arrows), red cells are CD30L+ cells (M–R) (red arrows), pink-brown cells are OX40L+/tryptase+ cells (A-J) and blue-red cells are CD30L+/c-Kit+ cells (M–R) (green arrows). Quantitative analysis of the % of OX40L+/tryptase+ MCs among all MCs (K), OX40L+/tryptase+ MCs interacting with CD8+ T-cells (L) and the % of CD30L+/c-Kit+ MCs among all MCs (S) in AA patients compared to controls. Analysis derived from 17–21 areas of 6–14 HFs of 6–7 healthy control and of 11–21 areas of 4–12 HFs of 3 AA patients for non-lesional skin and of 17–21 areas of 16–17 HFs of 7 AA patients for lesional skin. ±SEM ***p≤0.001, **p≤0.01, *p≤0.05 ±SEM, One-Way ANOVA or Kruskal-Wallis test followed respectively by Bonferroni's or Dunn's multiple comparison tests. Immunohistochemical identification of 4–1BBL+/c-Kit+ and 4-1BBL-/c-Kit+ MCs, detected using 4–1BBL/c-Kit/CD8 staining (T–W) and of ICAM-1+/tryptase+ and ICAM-1-/tryptase+ MCs, detected using ICAM-1/CD8/Tryptase staining (X-AA), around the HF bulb of AA patient (T,X) and control (W,Z). Representative pictures of 4–1BBL+/c-Kit+ (U), 4–1BBL-/c-Kit+ MCs (V), ICAM-1-/tryptase+ (Y) and ICAM-1+/tryptase+ (AA) MCs interacting with CD8+ T-cells. Brown cells are CD8+ (T–W) or ICAM-1+ (X-AA) cells (brown arrows), blue cells are c-Kit+ MCs (T–W) or CD8+ cells (X-AA) (blue arrows), red cells are 4–1BBL+ cells (T–W) (red arrows), pink cells are tryptase+ cells (X-AA) (pink arrows), blue-red cells are 4–1BBL+/c-Kit+ MCs (T–W) and pink-brown cells are ICAM-1+/tryptase+ MCs (X-AA) (green arrows). These stainings were observed in one section/subject of 6–8 healthy individuals and non-lesional skin from 4 AA patients and lesional skin from 11 AA patients. Scale bars: 20 µm (A–C, N–P,AA), 10 µm (D–E,U,V,Y), 5 µm (F–H), 50 µm (I–J,M,Q,R,T,W,X,Z). Connective tissue sheath (CTS), dermal papilla (DP), hair follicle (HF), hair matrix (HM), perifollicular dermis (PFD).

OX40L is a type II transmembrane soluble glycoprotein which activates OX40 during cell contact thereby stimulating CD8+ T-cell proliferation, survival, and cytokine production [76], [77], [80]–[85]. Quantitative (immuno-)histomorphometry showed that the total number of OX40L+ MCs was increased in lesional skin of AA patients (**Figure 4A–E,I–J**). In addition, the percentage of OX40L+ MCs among all tryptase+ MCs was significantly increased compared to non-lesional AA skin (**Figure 4I–K**). While most of the MCs interacting with CD8+ T-cells in all groups expressed OX40L, lesional AA showed a significant up-regulation of OX40L+ MCs that were in direct contact with CD8+ T-cells (**Figure 4G–H,L**).

Next, we also performed triple-IHC for CD30L, since its expression by MCs is up-regulated under pro-inflammatory conditions [86]–[88]; moreover, activated CD8+ T-cells can express CD30 which is implicated in the control of CD8+ T-cell proliferation and cytokine production [83], [85], [89]–[91]. **Figure 4M–S** shows that the total number and percentage of CD30L+ MCs was significantly up-regulated in lesional AA skin compared to healthy and non-lesional human skin (**Figure 4Q,S**). However, hardly any CD30L+ MCs were seen to be in contact with CD8+ T-cells (**Figure 4O,P**).

4–1BBL is expressed by activated MCs [35], [76], [92] and supports CD8+ T-cell survival/expansion after binding its receptor on activated T-cells [83], [93]–[98]. Triple-IHC revealed that perifollicular 4–1BBL+ cells are exceptionally rare in healthy and non-lesional AA skin (**Figure 4W**). However, more 4–1BBL+ MCs were detectable in lesional AA skin, notably in a peribulbar location (**Figure 4T–U**)**,** consistent with the typical peribulbar inflammatory infiltrate in AA [1], [2], [14], [65], and occasionally also very close to CD8+ T-cells (**Figure 4T–V**).

ICAM-1 IR was also examined, since ICAM+ MC-derived exosomes can induce T lymphocyte proliferation and cytokine production [99], [100]. Corresponding triple-IHC revealed a slight increase in the number of ICAM-1+ MCs in AA lesional skin (**Figure 4X,Z**). Yet, only a few ICAM-1+ MCs were seen to be located in close proximity to CD8+ T-cells (**Figure 4AA**).

Collectively, these results further support the concept that MCs in AA are skewed towards pro-inflammatory activities and that the OX40/OX40L, and possibly also the 4-1BB/4–1BBL and/or ICAM-1/LFA-1 signalling pathways might be involved in regulating abnormal MC-CD8+ T-cell interactions in AA.

### Immuno-inhibitory MCs appear to be defective in AA

We then asked whether immuno-inhibitory molecules are down-regulated on MCs in AA skin *in situ*, along with the reduced TGFβ1 expression of perifollicular MCs reported above (**Figure 2A–D**). First, IL-10 was assessed as MCs can regulate peripheral tolerance by releasing IL-10 [29], [30], [37], [49], [52], [54], [101], a predominantly immuno-inhibitory type II cytokine [102]–[104].

**Figure 5:**
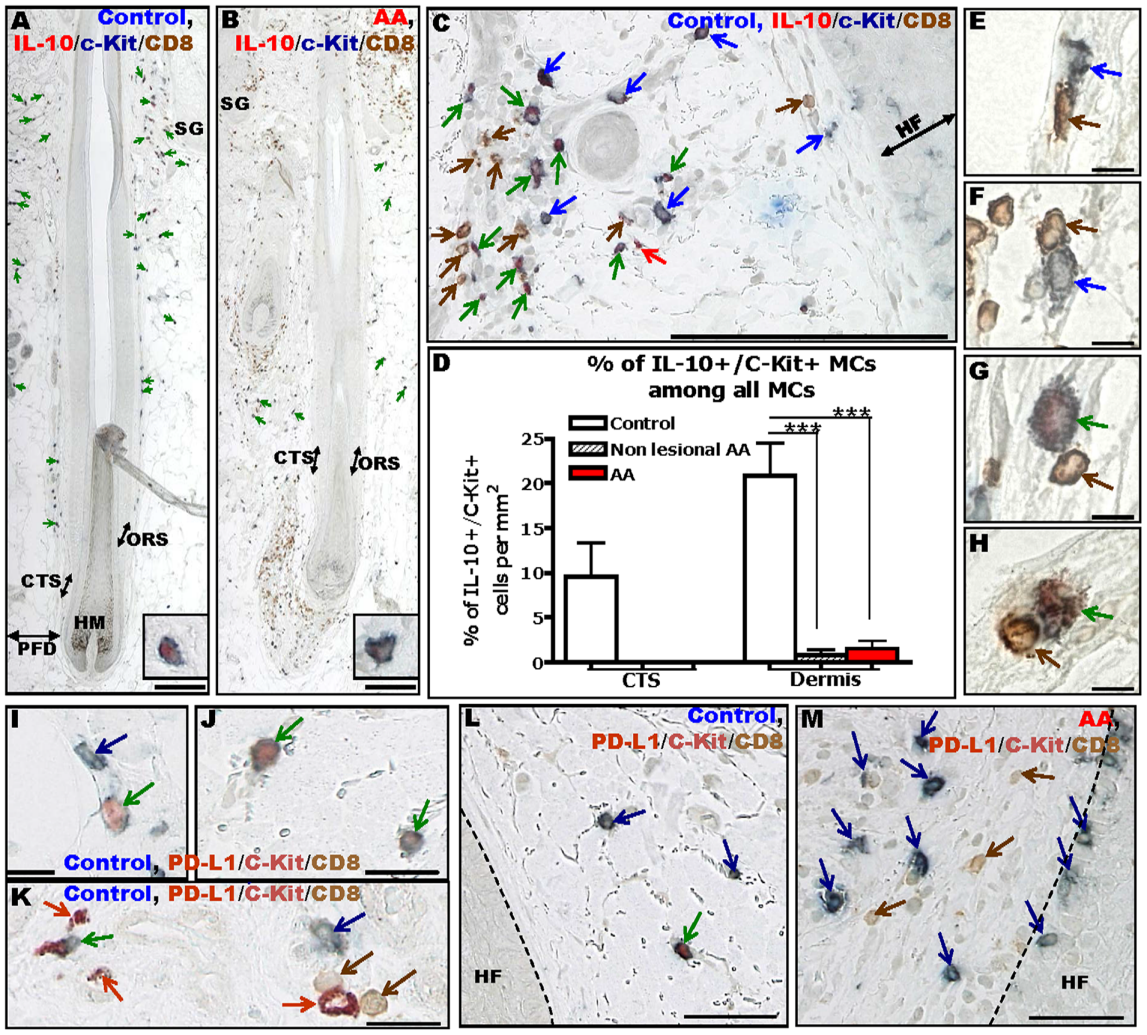
Immuno-inhibitory MCs and MC-CD8+ T-cell interactions appear to be down-regulated in AA. Immunohistochemical identification of c-Kit+/IL-10+ MCs and CD8+ T-cells in human scalp skin of controls (A) and AA patients (B), higher magnification of a single MCs in inserted panels. Higher magnification of IL-10/CD8/c-Kit triple IHC which shows c-Kit+ MCs (blue arrows), c-Kit+/IL-10+ MCs (green arrows), IL-10+ cells (red arrows) and CD8+ T-cells (brown arrows) (C). Quantitative analysis of IL-10+ MCs (detected by c-Kit) in AA patients compared to controls (D). Analysis derived from 20 areas of 11 HFs of 7 healthy controls and 14 areas of 5 HFs of 3 AA patients for non-lesional skin and 20 areas of 16 HFs of 7 AA patients for lesional skin, ***p≤0.001, ±SEM, Mann-Whitney-U-Test. Representative pictures of IL-10- (E,F) and IL-10+ (G,H) MCs (detected by c-Kit) interacting with CD8+ T-cells. Representative pictures showing low (I) and high (J) PD-L1 IR of human healthy skin MCs *in situ*. Immunohistochemical identification of PD-L1+/c-Kit+ MCs and CD8+ T-cells in human healthy skin (I-L) and AA patients (M). Brown arrows indicate CD8+ cells, blue arrows indicate c-Kit+ MCs, red arrows indicate PD-L1+ cells, green arrows indicate PD-L1+/c-Kit+ MCs. This staining was performed on one section/subject of 8 healthy individuals of non-lesional skin from 4 AA patients, and of lesional skin from 12 AA patients. Size bars: 200 µm (A–C), 50 µm (L,M), 20 µm (I,J) and 10 µm (E–H). Connective tissue sheath (CTS), hair follicle (HF), hair matrix (HM), outer root sheath (ORS), perifollicular dermis (PFD), sebaceous glands (SG).

Interestingly, most of the cells expressing prominent IL-10 IR in healthy human skin were MCs, primarily in the in CTS and PFD (**Figure 5A**). However, the number of IL-10+ MCs was significantly decreased in lesional and non-lesional AA skin compared to healthy controls (**Figure 5A–D**), and the few IL-10+ MCs which remained visible in AA patients were localized only rarely in the perifollicular mesenchyme (i.e CTS or PFD) (**Figure 5B**). Moreover, IL-10 expression in individual MCs was decreased in AA skin compared to healthy controls (**Figure 5A,B**, see higher magnification insert). Generally, MCs that interacted with CD8+ T-cells did not express substantial IL-10 IR, neither in healthy nor in AA skin (**Figure 5E–H**).

PD-L1 is a type I transmembrane protein implicated in HF-IP [105]. It delivers an inhibitory signal through its receptor on T-cells (PD-1), inhibiting cytokine production and proliferation while stimulating T-cell death [106], [107], [108], [109]. Here, we show the first evidence that primary human MCs can express varying levels of PD-L1 *in situ* in healthy human skin (**Figure 5I–L**), as has previously been shown for murine bone marrow derived-MCs [77]. In human healthy control skin, PD-L1+ MCs were rare (**Figure 5L**); their number appeared to be further reduced in lesional AA skin compared to healthy skin (**Figure 5L,M**). The number of PD-L1+ cells was too low to permit a quantitative analysis. Moreover, we could not detect any PD-L1+ MCs interacting with CD8+ T-cells in healthy or AA skin (data not shown).

The final functional MC marker we examined in this series of experiments was the important immuno-inhibitory “no danger-signal”, CD200, which plays a key role in HF-IP maintenance [8], [59], [110] and whose receptor is expressed on T-cells [111]. However, in line with a previous report [112], we could find almost no CD200+ MCs, neither in healthy human skin nor in AA lesional skin (data not shown).

Taken together, our observation that MCs in healthy skin express classical immuno-inhibitory molecules supports the hypothesis that, physiologically, perifollicular MCs mainly have tolerance-promoting functions [53]. Furthermore, MC expression of immuno-inhibitory proteins was reduced in AA skin, particularly during their interactions with CD8+ T-cells. This underscores that MCs in AA are skewed towards pro-inflammatory activities and that MC-CD8+ T-cell interactions in AA are predominantly pro-inflammatory.

Pilot experiments that attempted to functionally probe MC-CD8+ T-cells interaction in organ-cultured intact human scalp HFs or skin *in vitro* were inconclusive due to methodological constraints that could not be overcome (see Result S7 in File S1 for details).

### The number of MC-CD8+ T-cell contacts is also increased in C3H/HeJ mice affected by AA

**Figure 6:**
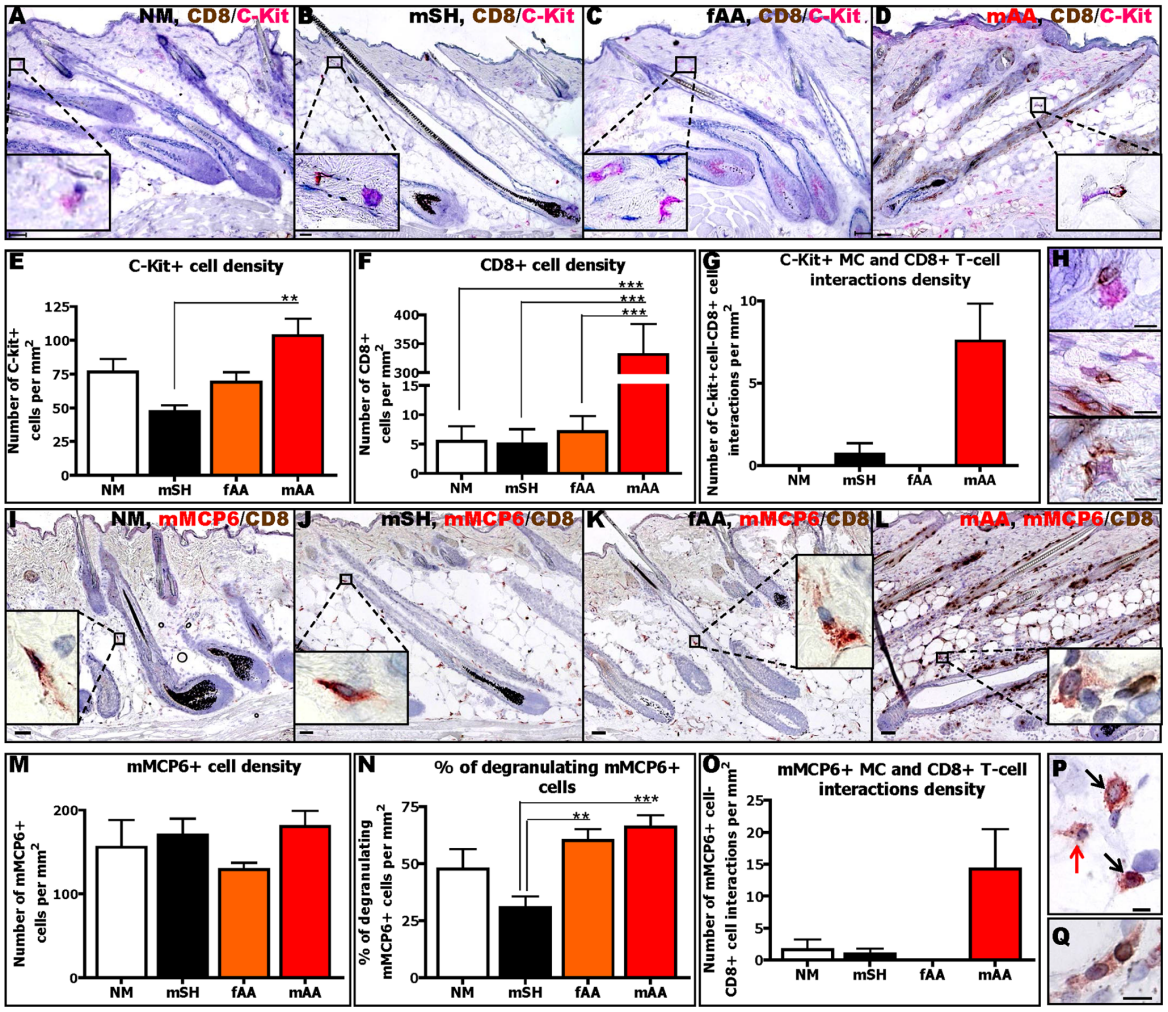
MC numbers, degranulation and interactions with CD8+ T-cells are increased in the C3H/HeJ-mouse-model of AA. Representative pictures of c-Kit/CD8 double-staining in normal (NM) (A), sham-grafted (mSH) (B), failed-grafted (fAA) (C) and AA (mAA) (D) mice. C-Kit+ MCs are labelled in pink while CD8+ T-cells are labelled in brown. Quantitative analysis of c-Kit+ (E), CD8+ T-cells (F) and c-Kit+ MC/CD8+ T-cell interactions (G) in mAA compared to NM, mSH and fAA control mice. Representative pictures of c-Kit+ MC-CD8+ T-cell interaction (H). Representative pictures of mMCP6/CD8 double-staining in normal (NM) (l), sham-grafted (mSH) (J), failed-grafted (fAA) (K) and AA (mAA) (L) mice. mMCP6+ MCs are labelled in red while CD8+ T-cells are labelled in brown. Quantitative analysis of mMCP6+ cells (M), % of degranulation (N) and mMCP6+ MC-CD8+ T-cell interactions (O) in mAA compared to NM, mSH, fAA control mice. Immunohistochemical identification of non-degranulating (black arrows) and degranulating (red arrow) of mMCP6+ MCs (P) and mMCP6+MC-CD8+ T-cell interaction (Q). Analysis derived from 6 HFs/mouse of 2–4 mice/group ±SEM, One-Way ANOVA or Kruskal-Wallis test followed by Bonferroni's test or Dunn's test (*p≤0.05, **p≤0.01, ***p≤0.001). Scale bars: 50 µm (A–D, M–L) and 10 µm (G,P,Q). AA mice (mAA), failed-grafted AA mice (fAA), normal mice (NM), sham-grafted mice (mSH).

Next we wished to probe whether abnormal MC-CD8+ T-cell interactions in human AA skin were also present in the C3H/HeJ mouse model for AA [55], [56]. As shown in **Figure 6A–Q**, normal (NM), sham-grafted (mSH) and failed-grafted (fAA) mice showed only a few perifollicular MCs (detected by c-Kit (**Figure 6A–C**) and mouse MC protease (mMCP) 6 (**Figure 6I–K**)) and very few CD8+ T-cells (**Figure 6A–C,I–K**). In striking contrast, lesional skin of mice affected by AA (mAA) showed significantly more perifollicular (immature) c-Kit+ MCs (**Figure 6A–E**), increased MC degranulation (**Figure 6I–L,N,P**) and higher CD8+ T-cell numbers (**Figure 6A–D,F,I–L**) than control mice. This was accompanied by increased MC-CD8+ T cell interactions, both of c-Kit+ (**Figure 6G–H**) and of mMCP6+ MCs (**Figure 6O,Q**). However, the total number of mature, mMCP6+ skin MCs remained essentially unaltered between the groups (**Figure 6I–M**). These data from AA mice independently suggest that abnormal MC activities and MC-CD8+ T-cell interactions are a general feature of the AA phenotype across species.

### MC-CD8+ T-cell interactions are also abnormal in experimentally induced human AA

During the course of these experiments, a novel humanized mouse model for AA research became available, in which healthy human scalp skin transplanted onto SCID mice is experimentally transformed into an AA phenotype [5], [57].

Therefore, we asked whether abnormal MC-CD8+ T-cell interactions can also be seen in AA lesions in human scalp skin induced by IL-2-treated PBMCs from healthy donors that were enriched for NKG2D+/CD56+ cells.

**Figure 7:**
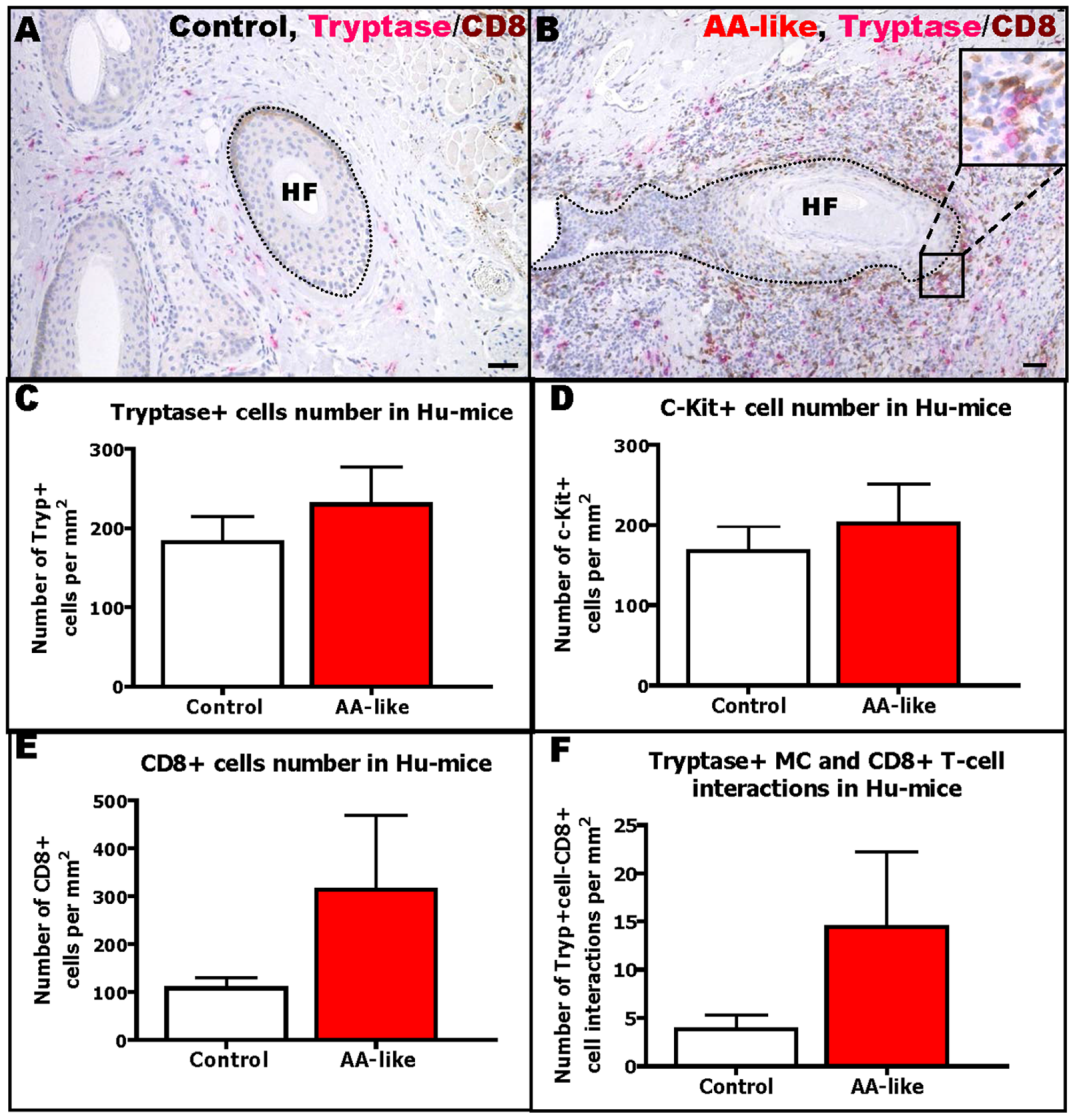
MC numbers and interactions with CD8+ T-cells are increased in the humanized-mouse model of AA. Representative pictures of tryptase/CD8 double-staining in control (A) and AA-like (B) mice. Tryptase+ MCs are labelled in pink while CD8+ cells are labelled in brown. Quantitative analysis of tryptase+ MCs (C), c-Kit+ MCs (D), CD8+ cells (E) and tryptase+ MC-CD8+ cell interactions (F) in AA-like mice compared to control mice. Analysis deriving from 1–6 areas (HFs)/mouse of 3 mice/group from 2 experiments, ±SEM, Student's t-test or Mann-Whitney-U-Test (ns). Scale bars: 50 µm.

Quantitative immunohistomorphometry confirmed that the experimentally induced AA lesions in transplanted human scalp skin show both more perifollicular CD8+ T-cells (**Figure 7B,E**), and more perifollicular MCs (**Figure 7B–D**) compared to human skin control transplants injected with PHA-treated PBMCs enriched for NKG2D+/CD56+ cells (**Figure 7A,C–E**). Moreover, the number of MCs in physical contact with CD8+ T-cells was higher in AA-like human skin lesions on SCID mice than in controls (**Figure 7A,B,F**). This further supports the hypothesis that abnormal MC-CD8+ T-cell interactions are functionally important for AA pathogenesis.

## Discussion

The concept that MCs are involved in the pathogenesis of AA dates back several decades [12], [14]–[16], but has still not been systematically followed-up. However, recent reports that anti-histamines may be beneficial in at least some AA patients [113]–[115] underscore the practical clinical relevance of dissecting the contribution of MCs to AA pathogenesis. Our results essentially confirm and then significantly extend the previous literature (e.g. [12], [14]–[16] (see Discussion S8 in File S1)) by focusing on MC interactions with CD8+ T-cells, the key immunocytes in AA pathogenesis [3]–[5], [14], [19]–[24].

We show that physical MC-CD8+ T-cell interactions, a fundamental prerequisite for CD8+ T-cell activation by MCs [35], [77], are enhanced and abnormal in the perifollicular mesenchyme of lesional AA skin in a) AA patients, b) AA mice, and in c) healthy human skin experimentally transformed into lesional AA skin, i.e. in both spontaneous and induced AA and across two mammalian species.

**Figure 8:**
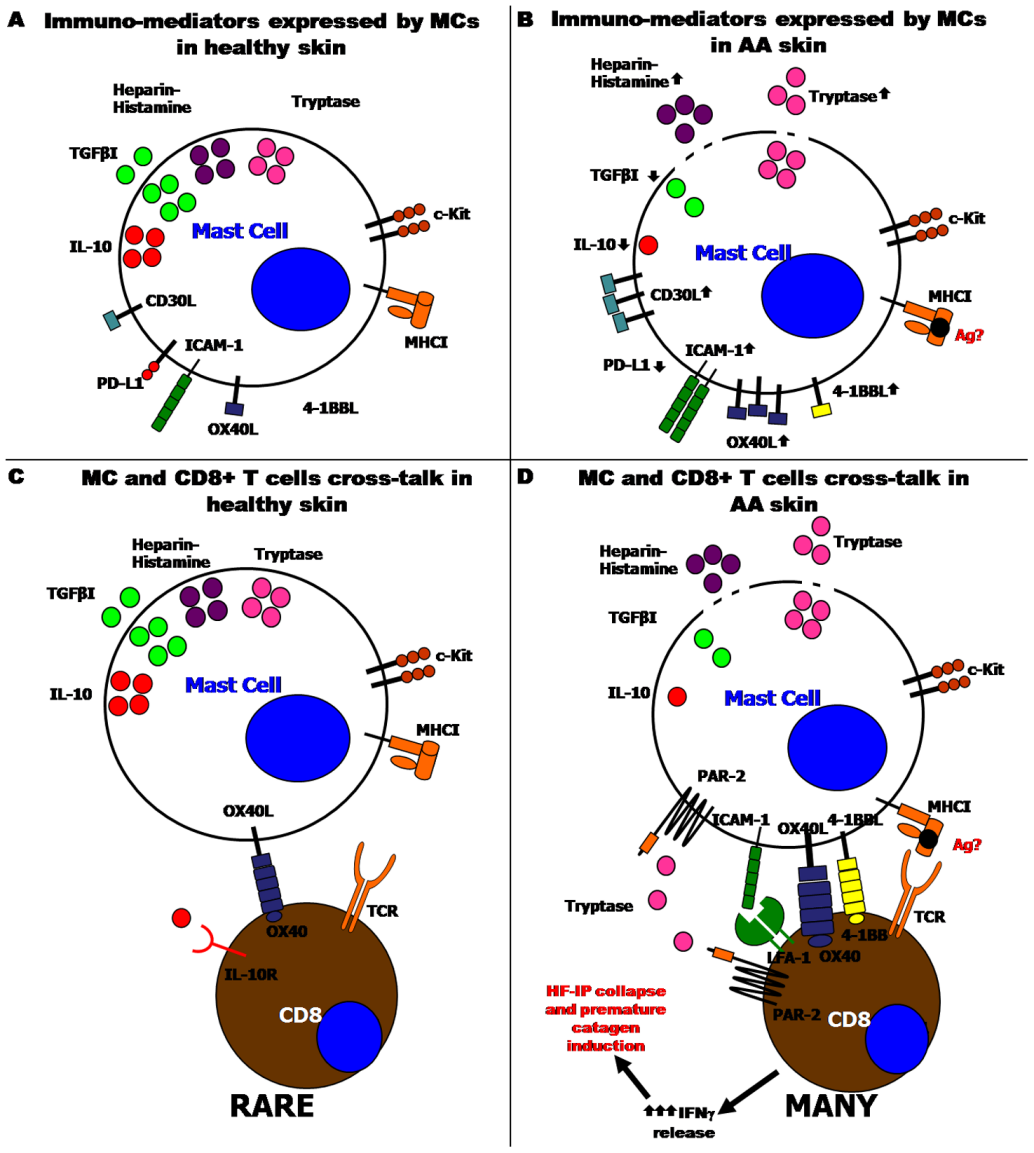
Schematic summary: MC immuno-phenotype and MC-CD8+ T-cell interactions in healthy compared to lesional AA skin. In human healthy skin, MCs are mostly non-degranulated and they express the SCF receptor, c-Kit, and MHCI molecules. Most of them express IL-10, TGFβ1 while only some express OX40L and PD-L1 and very few express CD30L and ICAM-1 (A). In lesional AA skin, the degranulation of MCs is increased (release of tryptase, heparin and histamine) and the expression of tryptase is increased, while the contents of TGFbeta1, IL-10 are decreased. Moreover, the numbers of OX40L+, CD30L+, 4-1BBL+ and ICAM-1+ MCs are up-regulated, while MCs positive for IL-10 and PD-L1 are down-regulated (B). In human healthy skin, rare MCs are found in close contact with CD8+ T-cells and most of them express OX40L. Therefore, we hypothesized that OX40/OX40L might mediate this interaction. Rarely, we found IL-10+ MCs interacting with CD8+ T-cells (C). In lesional AA skin, many MCs interact with CD8+ T-cells. During this cross-talk, most MCs express OX40L but instead, in some rare cases, 4-1BBL and ICAM-1 were expressed. These ligands might stimulate the activation and proliferation of CD8+ T-cells. Since MCs during the interaction are also degranulating, we hypothesize an activation of PAR-2 (tryptase receptor) on CD8+ T-cells. Finally, we suggest that MCs may operate as autoantigen-presenting cells (D). This schematic drawing was prepared using the Biomedical-PPT-Toolkit-Suite of Motifolio Inc., USA.

Moreover, we demonstrate that MCs switch from a protective immuno-inhibitory to a pro-inflammatory phenotype (**Figure 8** and Table S2 in File S1). This may promote pathogenic CD8+ T-cell responses against HFs (Discussion S9 in File S1). We envision that this MC phenotype switch could enhance CD8+ T-cell secretion of IFNγ [35], the recognized key cytokine in AA pathogenesis [1], [2], [10], [11]. Such a MC phenotype switch has already been reported in other autoimmune diseases [25], [26], [38], [87]. IFNγ is expected to trigger two key events in AA pathogenesis [1], [2]: the stimulation of premature HF regression (catagen) [116] and the induction of HF-IP collapse in humans and mice [11], [117]. **Figure 8** summarizes the main phenotypic differences that distinguish, according to our results, MCs in healthy human skin from those in lesional AA skin, and develops a plausible hypothetical scenario how abnormal MC-CD8+ T-cell interactions may promote AA pathogenesis.

Methodologically, the major shortcoming of the current study is its exclusively observational nature, despite its rigorous quantitative approach as well as the use of triple-IHC techniques and mutually complementary AA models. Satisfactory functional experiments to definitely confirm or refute the basic hypothesis proposed here (**Figure 8**) can currently not be performed for the following reasons: 1) None of the routinely used MC-deficient or MC-overexpressing mouse models develop classic AA lesions. 2) It is not yet possible to selectively eliminate or exclusively modulate *only* MCs *in vivo* (mice) or in transplanted or organ-cultured human skin *without also* eliminating or damaging other cutaneous cell populations (e.g. c-Kit+ melanocytes and hair matrix keratinocytes, or FceR+ Langerhans cells). 3) Successful cross-breeding of mice that spontaneously develop AA-like lesions (C3H/HeJ mice) with MC-deficient mouse strains [118] has not yet been achieved by any group. 4) Frequently employed inhibitors of MC degranulation such as cromolyn or luteolyn are not effective for all MC subsets and/or are not MC-specific, as other cell populations, including CD8+ T-cells and sensory neurons and their axons, are also affected [119]–[122].

Therefore, the concept that abnormal MC-CD8+ T-cell interactions play a functionally important role in AA pathogenesis, remains hypothetical and the underlying mechanisms of action can only be regarded as speculative on the basis of our phenomenological evidence – until the methodological handicaps summarized above have been overcome. Since the management of AA in clinical medicine remains overall quite unsatisfactory [1], [123] and in view of the psychoemotional stress this disease imposes on affected patients [124]–[126], it is a matter of urgency to develop more effective AA management strategies. Therefore, the phenomenological evidence presented here and the persuasive pathogenesis scenario deduced from it (**Figure 8**), are translationally important, since they help to identify novel candidate targets for future therapeutic intervention in AA.

Our findings predict that treatment regimens which promote an immuno-inhibitory phenotype and/or suppress the switch towards a pro-inflammatory MC phenotype, should down-regulate undesired CD8+ T-cell responses against human HFs. Our data suggest that it deserves to be tested in the two distinct AA mouse models employed here whether blocking OX40L/OX40 (e.g using oxelumab, which was first developed for the treatment of asthma [84], [127]) or 4-1BB/4-1BBL interactions [96], [128], or antagonizing PAR-2 [129], [130] affects the development of AA lesions and/or hair regrowth. However, as these signalling molecules are broadly expressed on immunocytes, more specific therapies should be contemplated, including the use of bi-specific antibodies so as to selectively block MCs [120]. In any case, our study underscores that the therapeutic modulation of perifollicular MCs in human skin merits systematic exploration as a novel therapeutic strategy in the future management of AA.

## Supporting Information

File S1 Background: S1–S2. Material and methods: S3. Result: S4–S7. Discussion: S8–S9. References. Figure S1. Positive controls for triple-immunostainings. Figure S2. MC density is significantly increased in lesional skin compared to non-lesional skin of AA patients and scalp skin from healthy subjects. The number of proliferating c-Kit+ MC is tendentially increased in AA lesional skin compared to control skin. Figure S3. The maximal increase of MC density is found in AA patients in subacute stage of the disease. Figure S4. TGFβ1 immunoreactivity is decreased in the HF ORS of AA patients. Figure S5. MHCI/CD8/Tryptase triple staining showing MHCI+ MCs in close contact with CD8+ T-cells. Table S1. Primary antibody. Table S2. Expression of pro-inflammatory and pro-inhibitory molecules and cytokines which are considered to be involved in the cross-talk between MCs and CD8+ T-cells in PFD of lesional AA skin compared to non-lesional AA and healthy skin.
(PDF)
