# Supplementary material for: Abnormal Interactions between Perifollicular Mast Cells and CD8+ T-Cells May Contribute to the Pathogenesis of Alopecia Areata
Source: PLoS One. 2014 May 15;9(5):e94260. doi: 10.1371/journal.pone.0094260 (PMC4022513; doi:10.1371/journal.pone.0094260)
Supplement: File S1 — Background: S1–S2. Material and methods: S3. Result: S4–S7. Discussion: S8–S9. References. Figure S1. Positive controls for triple-immunostainings. Figure S2. MC density is significantly increased in lesional skin compared to non-lesional skin of AA patients and scalp skin from healthy subjects. The number of proliferating c-Kit+ MC is tendentially increased in AA lesional skin compared to control skin. Figure S3. The maximal increase of MC density is found in AA patients in subacute stage of the disease. Figure S4. TGFβ1 immunoreactivity is decreased in the HF ORS of AA patients. Figure S5. MHCI/CD8/Tryptase triple staining showing MHCI+ MCs in close contact with CD8+ T-cells. Table S1. Primary antibody. Table S2. Expression of pro-inflammatory and pro-inhibitory molecules and cytokines which are considered to be involved in the cross-talk between MCs and CD8+ T-cells in PFD of lesional AA skin compared to non-lesional AA and healthy skin. (PDF) [file pone.0094260.s001.pdf]

FILE S1: Combined Supporting Information files

Supplementary background S1

Several studies have reported an increase of the number of peribulbar and perivascular mast cells (MCs) in alopecia areata (AA) lesions [12,14-16]. However, other authors did not find any differences in AA skin compared to controls with respect to MC histochemistry [62]. Thus, an as yet ill-defined role for MCs in AA has previously been speculated [12,14-16], their role in the pathobiology of AA remains quite unclear.

In mammalian skin, MCs are located in the dermal and subcutaneous tissue and are particularly prominent in the connective tissue sheath (CTS) of the hair follicle (HF) [s1], where they play a role in the regulation of HF cycling [40,41,43,44]. After stimulation with the endogenous MC secretagogues, substance P (SP) or corticotropin-releasing hormone, anagen HFs can be induced to prematurely enter into catagen, the regression phase of HF cycling [44,45,60,s2,s3]. Interestingly, premature entry into catagen is also a hallmark of AA [1,2]. In contrast, MCs have also been proposed to exert protective effects on the HF [12,16,53]. Other authors have hypothesized that histamine release by degranulating MCs in AA skin may inhibit the maturation of suppressor T lymphocytes [15]. Recently, it was shown that IL-10 deficient mice develop a form of alopecia that is MC-dependent [s4]. While this encourages one to systematically explore the role of MCs in human HF biology and AA pathology, their role remains controversial and obscure.

Supplementary background S2

In this study we have used several markers to analyze MCs. C-Kit (CD117) is the high-affinity tyrosine kinase receptor for stem cell factor (SCF) expressed on the surface of MCs, toluidine blue (TB) specifically stains for MC metachromatic granules such as heparin and histamine, while tryptase is a neutral protease contained in pre-formed MC granules. Since SCF is the most important cytokine for the development of MCs and their survival, c-Kit receptor is expressed on mature and immature MCs [45,47,s5,s6]. Therefore, c-Kit staining is detectable on almost all MCs while TB and tryptase stain only mature MCs [47,s6].

Supplementary material and methods S3

Skin biopsies derived from healthy human subjects, AA patients and the humanized AA mouse model [5, 57] were fixed in 4% formalin for at least 48 hours and embedded in paraffin, while harvested back skin from C3H/HeJ [55,56] mice was immediately snap frozen in liquid nitrogen, and processed for sectioning. Paraffin sections were deparaffinised and heated with either sodium-citrate or TRIS-EDTA buffers while cryosections were fixed in acetone for 10 min at -20°C.

For detection of c-Kit (CD117), 4 µm skin sections were immunostained following established protocols [58,59] by using a monoclonal rabbit anti-human c-Kit antibody (1:400, DAKO, Hamburg, Germany in DAKO antibody diluent) (Supplementary table S1), followed by a biotinylated secondary goat anti-rabbit antibody (1:200, Jackson Immunoresearch Laboratories (JIR), West Grove, PA, USA in DAKO antibody diluent). The reaction was developed using the peroxidase-based avidin-biotin complex (ABC-HRP, Vector Laboratories, Burlingame, CA, USA) method and peroxidase-chromogen 3,3'-diaminobenzidine (DAB). As

counterstain for c-Kit immunostaining, Mayer's hematoxylin (Merck, Darmstadt, Germany) was used **(Table 2)**.

For double or triple-immunostaining, skin sections were serially stained for each protein. Skin sections were incubated with the first primary antibody (Supplementary table S1) dissolved either in tris buffered saline (TBS), TBS and normal serum, antibody diluent (DAKO) or DCS LabLine Antikörper-Verdünnungspuffer (DCS, Innovative Diagnostik-Systeme, Hamburg, Germany) followed by a biotinylated secondary goat anti-mouse/goat anti-rabbit antibody (1:200, JIR or Beckman Coulter) and developed using either ABC-HRP and DAB or 3-amino-9-ethylcarbazole (AEC, Vector) as substrate **(Table 2)**. After blocking with either normal serum, normal serum plus bovine serum albumin (BSA), BSA or normal serum plus BSA and X-triton 0,5%, this was followed by immunostaining for the second primary antibody (Supplementary table S1). The skin sections were then incubated with an appropriate secondary antibody and the ABC-alkaline phosphatase (ABC-AP, Vector) detection system using either SIGMAFAST™ (Sigma) or Vector Blue® as substrate **(Table 2)**. In order to detect the third protein, after proper blocking, the third primary antibody (Supplementary table S1) was applied, followed by the incubation of the secondary antibody, which was detected either with ABC-HRP or ABC-AP and DAB, SIGMAFAST™ **(Table 2)**.

For mMCP6/CD8 double-immunostaining, the mMCP6 protein (Supplementary table S1) was detected by using Envision®-HRP (DAKO) [59], following the manufacturer´s protocol **(Table 2)**. As described before, skin sections were then incubated with a second primary antibody (CD8) (Supplementary table S1) which was detected by ABC-HRP and DAB as a substrate **(Table 2)**.

For CD200 triple staining, we used HRP conjugated donkey anti-goat (JIR) as a secondary antibody for goat anti-human CD200 therefore it was not necessary to use ABC-HRP **(Table 2)**.

In some cases, skin sections were finally incubated with either Mayer's hematoxylin (Merck) or Methyl green (DAKO) as a counterstain **(Table 2)**.

For double- [116] or triple-IF, as secondary antibodies goat anti-mouse/goat anti-rabbit IgG conjugated with fluorescein isothiocyanate (1:400, JIR, FITC), rhodamine (1:400, JIR) or Dy Light 350 (1:50, Thermo Scientific) were used. Counterstaining of nuclei was achieved with DAPI (4',6-diamidine-2'-phenylindoldihydrochloride, Boehringer Mannheim, Germany) **(Table 2)**.

Supplementary results S4

Comparing the different staining methods, c-Kit immunostaining detected significantly higher numbers of MCs than tryptase IHC or TB histochemistry and showed the strongest increase of MC density in the PFD area, whereas the other two methods visualised higher MC numbers in the CTS **(Figure 1H)**. This was also seen by c-Kit/tryptase double-IF **(Figure 1G)**. MC increase in AA in different specific skin compartments (upper dermis, dermis and subcutis), was slightly variable when comparing the different staining methods (data not shown).

In order to analyse if the MC increase in AA compared to controls is localized only around HFs, we evaluated the number of tryptase+ MCs using Ki-67/tryptase IHC in one extra area demarcated 200µm to 400µm from the basement membrane of the HFs. The analysis revealed no change in MC density between this extra area and PFD (data not shown).

Supplementary result S5

HF-IP is collapsed in AA patients

Since AA pathogenesis is characterized by a collapse of the IP of anagen hair bulbs [1,2,6,9], we investigated whether this is also seen in the AA skin samples examined in the current study by analysing intrafollicular TGFβ1 protein expression, one of the chief guardians of HF-IP [1,6-9,69]. TGFβ1 immunoreactivity (IR) showed the expected strong expression in the HF ORS of healthy skin, as well as in some perifollicular cells, including MCs (Supplementary Figure S4A-B). Quantitative (immuno-)histomorphometry revealed a strong diminution of TGFβ1 IR in the ORS of lesional AA HFs (Supplementary Figure S4A-C).

Supplementary result S6

AA MCs show prominent MHC class I immunoreactivity while interacting with CD8+ T-cells

MHC class I is expressed in all nucleated cells of the human body, apart from IP sites [1,7-9]. MCs are able to present autoantigens to CD8+ T-cells via MHC class I and can drive and control CD8+ T-cell-dependent immune responses [35]. Here, perifollicular MCs strongly expressed MHC class I in lesional human AA skin, also when they physically interacted with CD8+ T-cells (Supplementary Figure S5A-D). Therefore, it is conceivable that MCs may operate as autoantigen-presenting cells in AA.

Supplementary result S7

Organ culture experiments do not allow one to functionally probe MC-CD8+ T-cell interactions in situ

In order to probe the effects of MC secretagogues on MC-CD8+ T-cell interactions, human HF organ culture was performed as described [s2], treating HFs with SP (10^{-8}M and 10^{-10}M). However the total number of detectable CD8+ T-cells in the HF's CTS was so low under the assay conditions (n = 4 positive cells in 26 HFs analysed) that their interactions with MCs could not be meaningfully investigated. Therefore, full thickness human scalp skin organ culture [s7] was employed as an alternative method, which contained higher numbers of detectable CD8+ T-cells (n = 9.26±1.98 positive cells/mm^2 after 3 day culture in vehicle group). Healthy human scalp skin was treated with the endogenous MC secretagogue, SP (10^{-8}M and 10^{-10}M), or the exogenous standard secretagogue, compound 48/80 (5µg/µl). This experiment was repeated twice, using skin fragments from three distinct individuals, including increasing the concentration of compound 48/80 to 50µg/µl. In one patient, we found that SP indeed increased the number of CD8+ (vehicle: 9.21±1.98, SP10^{-8}M: 20.72±4.69 positive cells/mm^2) and tryptase+ (vehicle: 46.26±2,94, SP10^{-8}M: 58.10±4,88 positive cells/mm^2) perifollicular cells. However, again, the frequency of detectable MC-CD8+ T-cell contacts (0.53±0.2 complexes/mm^2 after 3 days of culture in vehicle group) was too low to obtain significant results.

For obvious reasons, large AA skin biopsies needed for organ culture are essentially unobtainable and are ethically difficult to justify. However, we had the unique opportunity of obtaining, with written consent, a larger strip of alopecic scalp skin for full thickness skin organ culture from a female patient (age 67) with long-standing AA totalis (duration >10 years) who underwent cosmetic facelift surgery. Due to the long duration of the disease, only very few miniaturized HFs and a very discrete inflammatory cell infiltrate were seen, as expected from the literature [65], and the

infiltrate was likely further artificially reduced by loss of immunocytes after 3 days of organ-culture. However, treatment with SP showed a (non-significant) tendency towards increased MC interactions with CD8+ T-cells (vehicle: 1.51±0.74, SP10^{-10}M: 5.2±2.24, SP10^{-8}M: 1.28±0.7 complexes/mm^2) while no effect was seen with compound 48/80 (1.48±.0,58 complexes/mm^2). Slightly decreased MC interactions with CD8+ T-cells were observed after treatment with cromoglycate (10^{-7}M: 0.66±0.47 and 10^{-4}:0.21±0.2M respectively).

Supplementary discussion S8

Confirming previous observations [12,14-16], we found not only a significant MC density increase in AA patients compared to control skin **(Figure 1H)**, but also an up-regulation of MC proliferation **(Figure 1O)** and degranulation **(Figure 1P)**. The slight, but conspicuous difference between the staining methods is likely to be explained by the different MC markers that were targeted [45,47,s5,s6]. Moreover, the fact that significantly more c-Kit+ than TB+ and tryptase+ MCs were detected **(Figure 1H)**, suggests that the skin of AA patients shows a relative increase in the percentage of immature (c-Kit+/TB-/tryptase-) MCs. This is corroborated by our observation of the increased MC proliferation in AA patients, which was proportional to the up-regulation of mature MCs. Moreover, since MCs are able to differentiate from MC precursors also without proliferation [45-47], this would explain the increase of mature MCs in AA. At the same time, TB histochemistry revealed more positive cells than tryptase immunostaining **(Figure 1H)**. This was expected from the much broader specificity of the histochemical technique, which detects multiple different MC granule contents (incl. heparin and histamine) as opposed to monospecific tryptase IHC. With both techniques one has to keep in mind that these granule-dependent markers may fail to demarcate anaphylactically

degranulated MCs since the degranulated state can last for hours until subsequent granule re-synthesis [44,47,s5,s8,s9].

Considering the immediate release of pre-formed pro-inflammatory proteins during MC degranulation [29,58], it is interesting to note that the percentage of degranulating MCs in AA patients was significantly up-regulated compared to the controls **(Figure 1P)**. The massive release of pre-formed MC granules containing e.g. neutral proteases (tryptase, chymase), histamine, proteoglycans (heparin and chondroitin sulphate E) and cytokines like TNF-α [26,29,30,32,36,44,54,73-75,77,s10], together with the substantial increase in the total number of MCs in lesional AA skin **(Figure 1H)**, is expected to create a strongly pro-inflammatory perifollicular microenvironment which may promote the pathogenesis cascade leading to the AA phenotype [2].

Supplementary discussion S9

In the following, we briefly discuss the MC mediators examined here in regards to CD8+ T-cells and their potential role in AA pathogenesis.

Apart from stimulating CD8+ T-cells [30,s11], MC derived tryptase **(Figure 1C,F,I,J,M,O-P and 2E-I)** in AA could play a role in MC-directed collagenolysis [31,72,s12], leading to a disruption of the HF basement membrane; this could facilitate immigration of immunocytes into the – normally relatively shielded – HF epithelium, and could activate CD8+ T-cells. Moreover, it can promote the release of immune-mediators from keratinocytes [30,s13] and/or enhance MC activation [30,70,s14]. A key role for MC-dependent neurogenic inflammation is now well-appreciated in psoriasis [30,s15,s16] and may also apply to AA. Tryptase also

elicits action potentials in sensory skin nerves [30,s10,s17]. This may be particularly relevant in the context of MC-dependent neurogenic skin inflammation [s18,s19], as psychoemotional stress-induced neurogenic inflammation is increasingly viewed as a notable contributing factor in AA pathogenesis [126,s2,s3,s18,s19].

Confirming previous *in vitro* results obtained for cultured human and mouse MCs [76,77,s20,s21,s22,s23] and in chronic GVHD [s24], here we show that MCs can express OX40L (syn: CD252, CD134L) in human healthy and AA skin *in situ* **(Figure 4A-J)**. While OX40L can be found in the nucleus, cytoplasm or cell membrane of human skin MCs, eventually, OX40L becomes preferentially localized at one side of the cells, thus facilitating juxtacrine signalling [80]. As shown in **Figure 4A**, this phenomenon was also seen in human skin MCs. OX40L expression on the surface of MCs may therefore facilitate MC-CD8+ T-cell interactions in both healthy human and AA skin, since OX40L appeared to be the most prominently expressed co-stimulatory molecule on MCs in physical contact with CD8+ T-cells **(Figure 4L)**. Given that OX40L+ MCs can enhance T-cell activation, proliferation, survival and cytokine production *in vitro* [27,29,76,77,80,81,s23], OX40L-OX40 interactions may be important in modulating MC-CD8+ T-cell contacts not only in AA, but also in healthy skin.

The up-regulation of the number of CD30L+ (syn: CD153, TNFSF8) MC found here in AA skin **(Figure 4Q-S)** has already been reported in several pathological conditions, such as Hodgkin lymphoma [88], cancer [86], atopic dermatitis and psoriasis [87]. The latter observation is interesting since psoriasis shares some features with AA, e.g. both represent Th1-mediated inflammatory processes, and

both psoriasis [s25,s26] and AA may exhibit a Th17 phenotype [19,s27,s28]. Although CD30-CD30L interactions provide a co-stimulatory signal for T-cells and stimulate T-cell proliferation and cytokine production [83,85,89-91], we almost never found CD30L+ MCs in close contact with CD8+ T-cells in AA **(Figure 5O-P)**. This suggests that CD30L expression in MCs in AA might mediate their interaction with other immune cells (e.g. CD4+ T-cells) or with sCD30 [s29].

4-1BBL (syn.: CD137L, TNFSF9) enhances survival, proliferation, memory and cytolytic activities of T-cells and augments Th1- immune responses [93,94,96-98] and co-stimulated MC antigen presentation to CD8+ T-cells, at least in mice [35]. Therefore, despite the relative paucity of 4-1BBL+ MCs interacting with CD8+ T-cells **(Figure 5T-U),** these rare events could be very important for AA pathogenesis. In addition, the expression of 4-1BB is induced only after TCR activation, thus maintaining primed CD8+ T-cells after the inciting antigen is no longer available [96]. Therefore, our limited protein expression *in situ* data are well in line with the concept that the 4-1BB/4-1BBL pathway may be important regulating MCs and CD8+ T-cells in AA in pathogenesis.

Considering that only few data are available regarding the interactions of ICAM-1+ MC and LFA-1+ T-cells [100,s30,s31,s32] and most of these studies only consider the interaction of activated LFA-1+ T-cells with ICAM-1+ MCs [s30,s31,s32], our results that ICAM-1+MCs are interacting with CD8+ T-cells **(Figure 4 X-AA)** contribute to closing this research gap.

Supplementary References:

s1. Christoph T, Muller-Rover S, Audring H, Tobin DJ, Hermes B, et al. (2000) The human hair follicle immune system: cellular composition and immune privilege. Br J Dermatol 142: 862-873.

s2. Peters EM, Liotiri S, Bodo E, Hagen E, Biro T, et al. (2007) Probing the effects of stress mediators on the human hair follicle: substance P holds central position. Am J Pathol 171: 1872-1886.

s3. Siebenhaar F, Sharov AA, Peters EM, Sharova TY, Syska W, et al. (2007) Substance P as an immunomodulatory neuropeptide in a mouse model for autoimmune hair loss (alopecia areata). J Invest Dermatol 127: 1489-1497.

s4. Vanderford DA, Greer PK, Sharp JM, Chichlowski M, Rouse DC, et al. (2010) Alopecia in IL-10-deficient mouse pups is c-kit-dependent and can be triggered by iron deficiency. Exp Dermatol 19: 518-526.

s5. Sugawara K, Zakany N, Hundt T, Emelianov V, Tsuruta D, et al. (2013) Cannabinoid receptor 1 controls human mucosal-type mast cell degranulation and maturation in situ. J Allergy Clin Immunol 132: 182-193.

s6. Taketomi Y, Ueno N, Kojima T, Sato H, Murase R, et al. (2013) Mast cell maturation is driven via a group III phospholipase A2-prostaglandin D2-DP1 receptor paracrine axis. Nat Immunol 14: 554-563.

s7. Lu Z, Hasse S, Bodo E, Rose C, Funk W, et al. (2007) Towards the development of a simplified long-term organ culture method for human scalp skin and its appendages under serum-free conditions. Exp Dermatol 16: 37-44.

s8. Dvorak AM (2002) Ultrastructure of human mast cells. Int Arch Allergy Immunol 127: 100-105.

s9. Hammel I, Lagunoff D, Galli SJ (2010) Regulation of secretory granule size by the precise generation and fusion of unit granules. J Cell Mol Med 14: 1904-1916.

s10. Spinnler K, Frohlich T, Arnold GJ, Kunz L, Mayerhofer A (2011) Human tryptase cleaves pro-nerve growth factor (pro-NGF): hints of local, mast cell-dependent regulation of NGF/pro-NGF action. J Biol Chem 286: 31707-31713.

s11. Li T, He S (2006) Induction of IL-6 release from human T cells by PAR-1 and PAR-2 agonists. Immunol Cell Biol 84: 461-466.

s12. Krejci-Papa NC, Paus R (1998) A novel in-situ-zymography technique localizes gelatinolytic activity in human skin to mast cells. Exp Dermatol 7: 321-326.

s13. Lohi J, Harvima I, Keski-Oja J (1992) Pericellular substrates of human mast cell tryptase: 72,000 dalton gelatinase and fibronectin. J Cell Biochem 50: 337-349.

s14. Moormann C, Artuc M, Pohl E, Varga G, Buddenkotte J, et al. (2006) Functional characterization and expression analysis of the proteinase-activated receptor-2 in human cutaneous mast cells. J Invest Dermatol 126: 746-755.

s15. Balato A, Lembo S, Mattii M, Schiattarella M, Marino R, et al. (2012) IL-33 is secreted by psoriatic keratinocytes and induces pro-inflammatory cytokines via keratinocyte and mast cell activation. Exp Dermatol 21: 892-894.

s16. Hunter HJ, Griffiths CE, Kleyn CE (2013) Does psychosocial stress play a role in the exacerbation of psoriasis? Br J Dermatol 169: 965-974.

s17. Steinhoff M, Neisius U, Ikoma A, Fartasch M, Heyer G, et al. (2003) Proteinase-activated receptor-2 mediates itch: a novel pathway for pruritus in human skin. J Neurosci 23: 6176-6180.

s18. Kim HM, Lim YY, Kim MY, Son IP, Kim DH, et al. (2013) Inhibitory effect of tianeptine on catagen induction in alopecia areata-like lesions induced by ultrasonic wave stress in mice. Clin Exp Dermatol 38: 758-767.

s19. Zhang X, Yu M, Yu W, Weinberg J, Shapiro J, et al. (2009) Development of alopecia areata is associated with higher central and peripheral hypothalamic-pituitary-adrenal tone in the skin graft induced C3H/HeJ mouse model. J Invest Dermatol 129: 1527-1538.

s20. Fujita T, Kambe N, Uchiyama T, Hori T (2006) Type I interferons attenuate T cell activating functions of human mast cells by decreasing TNF-alpha production and OX40 ligand expression while increasing IL-10 production. J Clin Immunol 26: 512-518.

s21. Gri G, Piconese S, Frossi B, Manfroi V, Merluzzi S, et al. (2008) CD4+CD25+ regulatory T cells suppress mast cell degranulation and allergic responses through OX40-OX40L interaction. Immunity 29: 771-781.

s22. Nakano N, Nishiyama C, Yagita H, Koyanagi A, Akiba H, et al. (2009) Notch signaling confers antigen-presenting cell functions on mast cells. J Allergy Clin Immunol 123: 74-81 e71.

s23. Sibilano R, Frossi B, Suzuki R, D'Inca F, Gri G, et al. (2012) Modulation of FcepsilonRI-dependent mast cell response by OX40L via Fyn, PI3K, and RhoA. J Allergy Clin Immunol 130: 751-760 e752.

s24. Kotani A, Hori T, Fujita T, Kambe N, Matsumura Y, et al. (2007) Involvement of OX40 ligand+ mast cells in chronic GVHD after allogeneic hematopoietic stem cell transplantation. Bone Marrow Transplant 39: 373-375.

s25. Ariza ME, Williams MV, Wong HK (2013) Targeting IL-17 in psoriasis: from cutaneous immunobiology to clinical application. Clin Immunol 146: 131-139.

s26. Girolomoni G, Mrowietz U, Paul C (2012) Psoriasis: rationale for targeting interleukin-17. Br J Dermatol 167: 717-724.

s27. Lew BL, Cho HR, Haw S, Kim HJ, Chung JH, et al. (2012) Association between IL17A/IL17RA Gene polymorphisms and susceptibility to alopecia areata in the Korean population. Ann Dermatol 24: 61-65.

s28. Tojo G, Fujimura T, Kawano M, Ogasawara K, Kambayashi Y, et al. (2013) Comparison of interleukin-17- producing cells in different clinical types of alopecia areata. Dermatology 227: 78-82.

s29. Velasquez SY, Garcia LF, Opelz G, Alvarez CM, Susal C (2013) Release of soluble CD30 after allogeneic stimulation is mediated by memory T cells and regulated by IFN-gamma and IL-2. Transplantation 96: 154-161.

s30. Brill A, Baram D, Sela U, Salamon P, Mekori YA, et al. (2004) Induction of mast cell interactions with blood vessel wall components by direct contact with intact T cells or T cell membranes in vitro. Clin Exp Allergy 34: 1725-1731.

s31. Inamura N, Mekori YA, Bhattacharyya SP, Bianchine PJ, Metcalfe DD (1998) Induction and enhancement of Fc(epsilon)RI-dependent mast cell degranulation following coculture with activated T cells: dependency on ICAM-1- and leukocyte function-associated antigen (LFA)-1-mediated heterotypic aggregation. J Immunol 160: 4026-4033.

s32. Nagai K, Takahashi Y, Mikami I, Fukusima T, Oike H, et al. (2009) The hydroxyflavone, fisetin, suppresses mast cell activation induced by interaction with activated T cell membranes. Br J Pharmacol 158: 907-919.

s33. Shikotra A, Choy DF, Ohri CM, Doran E, Butler C, et al. (2012) Increased expression of immunoreactive thymic stromal lymphopoietin in patients with severe asthma. J Allergy Clin Immunol 129: 104-111 e101-109.

s34. Phillips TA, Ni J, Hunt JS (2003) Cell-specific expression of B lymphocyte (APRIL, BLyS)- and Th2 (CD30L/CD153)-promoting tumor necrosis factor superfamily ligands in human placentas. J Leukoc Biol 74: 81-87.

s35. Zhao S, Zhang H, Xing Y, Natkunam Y (2013) CD137 ligand is expressed in primary and secondary lymphoid follicles and in B-cell lymphomas: diagnostic and therapeutic implications. Am J Surg Pathol 37: 250-258.

s36. Goval JJ, Greimers R, Boniver J, de Leval L (2006) Germinal center dendritic cells express more ICAM-1 than extrafollicular dendritic cells and ICAM-1/LFA-1 interactions are involved in the capacity of dendritic cells to induce PBMCs proliferation. J Histochem Cytochem 54: 75-84.

s37. Poindexter NJ, Sahin A, Hunt KK, Grimm EA (2004) Analysis of dendritic cells in tumor-free and tumor-containing sentinel lymph nodes from patients with breast cancer. Breast Cancer Res 6: R408-415.

s38. Kshirsagar SK, Alam SM, Jasti S, Hodes H, Nauser T, et al. (2012) Immunomodulatory molecules are released from the first trimester and term placenta via exosomes. Placenta 33: 982-990.

s39. Lee EJ, Xu L, Kim GH, Kang SK, Lee SW, et al. (2012) Regeneration of peripheral nerves by transplanted sphere of human mesenchymal stem cells derived from embryonic stem cells. Biomaterials 33: 7039-7046.

s40. Delfortrie S, Pinte S, Mattot V, Samson C, Villain G, et al. (2011) Egfl7 promotes tumor escape from immunity by repressing endothelial cell activation. Cancer Res 71: 7176-7186.

s41. Shi F, Shi M, Zeng Z, Qi RZ, Liu ZW, et al. (2011) PD-1 and PD-L1 upregulation promotes CD8(+) T-cell apoptosis and postoperative recurrence in hepatocellular carcinoma patients. Int J Cancer 128: 887-896.

s42. Garza LA, Yang CC, Zhao T, Blatt HB, Lee M, et al. (2011) Bald scalp in men with androgenetic alopecia retains hair follicle stem cells but lacks CD200-rich and CD34-positive hair follicle progenitor cells. J Clin Invest 121: 613-622.

s43. Paus R, van der Veen C, Eichmuller S, Kopp T, Hagen E, et al. (1998) Generation and cyclic remodeling of the hair follicle immune system in mice. J Invest Dermatol 111: 7-18.

s44. Shin K, Watts GF, Oettgen HC, Friend DS, Pemberton AD, et al. (2008) Mouse mast cell tryptase mMCP-6 is a critical link between adaptive and innate immunity in the chronic phase of Trichinella spiralis infection. J Immunol 180: 4885-4891.

Supplementary Figure S1. Positive controls for triple-immunostainings

Specific single OX40L staining (A) showing OX40L+ activated T lymphocytes in the marginal zone of tonsil follicles [s33] and triple OX40L/CD8/Tryptase staining (B). Blue arrows indicate CD8+ T-cells, pink arrows indicate tryptase+ cells and green arrows indicate OX40L+/tryptase+ MCs. CD30L is expressed in human placental villous endothelial cells [s34], a positive control (C), along with tonsil sections (D) for the triple-staining CD30L/c-Kit/CD8. Red arrows indicate CD30L+ cells, blue arrows indicate c-Kit+ MCs, brown arrows indicate CD8+ T-cells and green arrows indicate CD30L+/c-Kit+ MCs. 4-1BBL+ cells are found in the marginal zone of human tonsil follicles [s35] (E), as shown also by triple 4-1BBL/c-Kit/CD8 immunostaining (F). Red arrows indicate 4-1BBL+ cells, blue arrows c-Kit+ MCs, brown arrows CD8+ T-cells, and green arrows 4-1BBL+/c-Kit+ MCs. Specific single staining for ICAM-1 (G) showing ICAM-1 immunoreactivity in the germinal centre [s36] and sparse positive cells of human tonsils (H), our positive control for ICAM-1/CD8/Tryptase triple-immunostaining. Brown arrows indicate ICAM-1+ cells, blue arrows CD8+ T-cells, and pink arrows tryptase+ cells. Many IL-10+ cells are found in tonsil sections [s37] as shown in our single IL-10 (I) but also triple IL-10/c-Kit/CD8 (J) stainings. Red arrows indicate IL-10+ cells, blue arrows indicate c-Kit+ MCs, brown arrows indicate CD8+ T-cells and green arrows indicate IL-10+/c-Kit+ MCs. PD-L1 immunoreactivity in chorionic villi of placenta [s38] detected by single PD-L1 (K) and triple PD-L1/c-Kit/CD8 (L) stainings. Red arrows indicate PD-L1+ cells, blue arrows indicate c-Kit+ MCs, brown arrows indicate CD8+ T-cells and green arrows indicate PD-L1+/c-Kit+ MCs. Positive CD200+ cells are found in the

HF bulge [8,59] (M), also for the triple staining CD200/CD8/Tryptase (N). Brown arrows indicate CD200+ cells, pink arrows indicate tryptase+ cells, blue arrows indicate CD8+ cells.

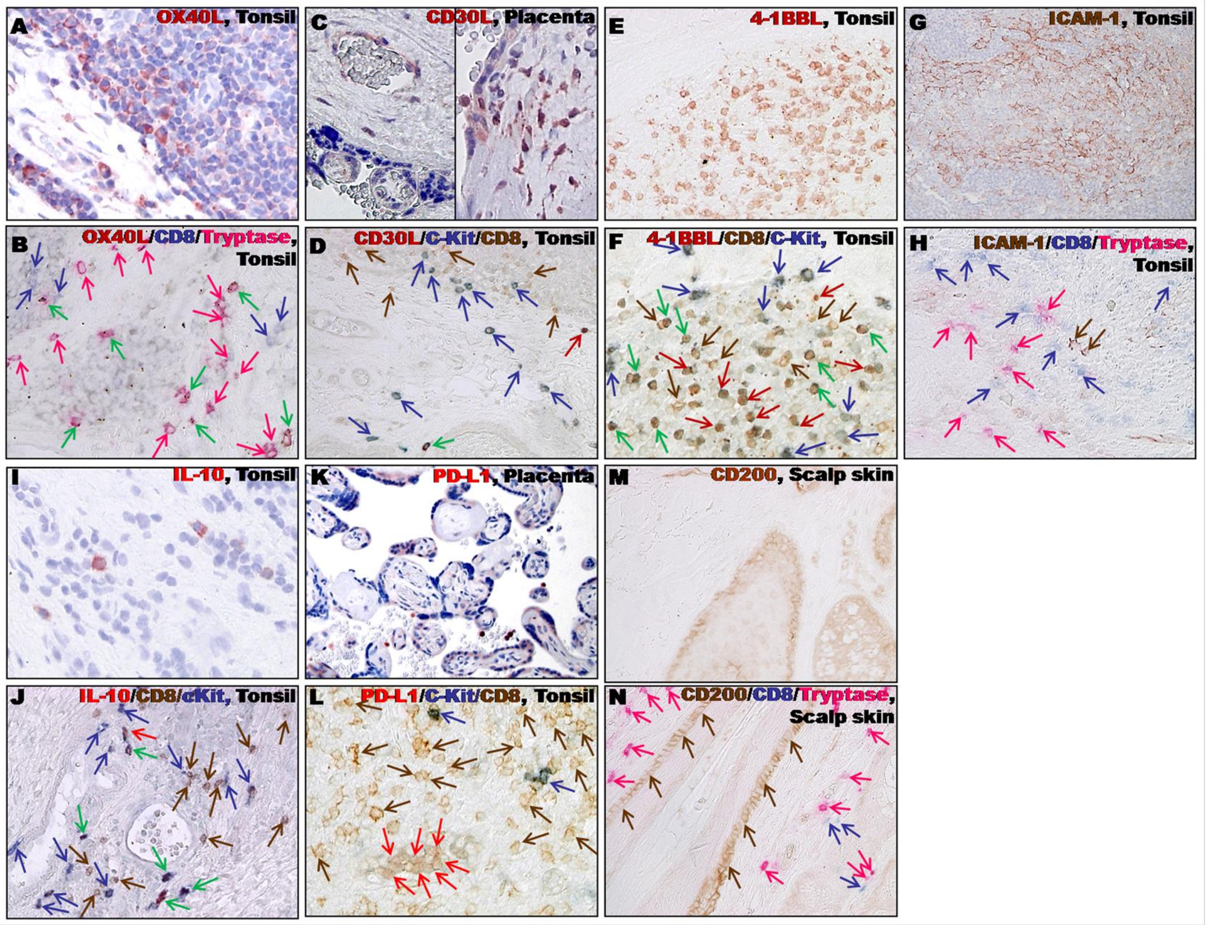

Supplementary Figure S2. MC density is significantly increased in lesional skin compared to non-lesional skin of AA patients and scalp skin from healthy subjects. The number of proliferating c-Kit+ MC is tendentially increased in AA lesional skin compared to control skin.

Quantitative analysis of the tryptase+ MCs (analysed using OX40L/CD8/tryptase) in lesional AA compared to non-lesional skin from AA patients and scalp skin from healthy subjects. Analysis derived from 17-21 areas of 6-14 HFs of 6-7 healthy controls and of 11-21 areas of 4-12 HFs of 3 AA patients for non-lesional skin and of 17-21 areas of 16-17 HFs of 7 AA patients for lesional skin (A). ***p≤0.001,

*p≤0.05 ±SEM, One-Way ANOVA followed respectively by Bonferroni´s multiple comparison tests.

Quantitative analysis of MC proliferation by Ki-67/c-Kit IHC (B). Analysis deriving from 31 areas of 30 HFs of 10 AA patients and 35 areas of 35 HFs of 7 healthy controls, ±SEM, Mann-Whitney-U-Test (ns).

Connective tissue sheath (CTS), perifollicular dermis (PFD).

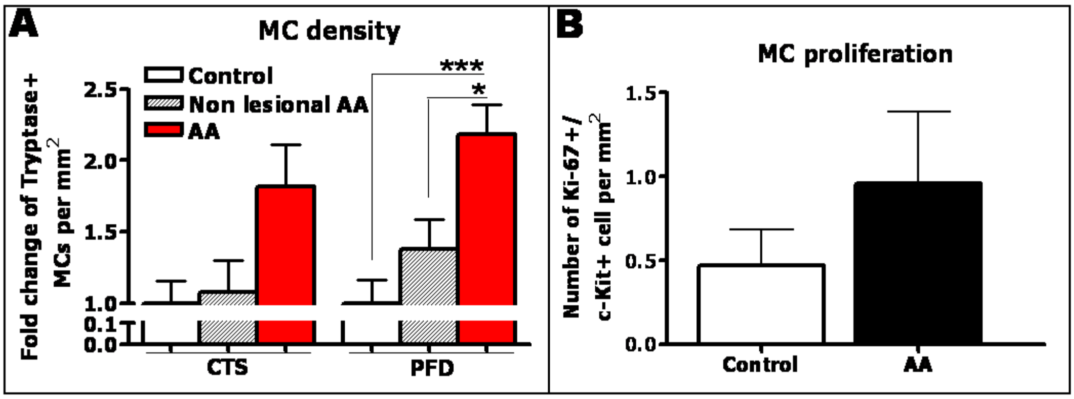

Supplementary Figure S3. The maximal increase of MC density is found in AA patients in the subacute stage of disease.

Representative pictures showing tryptase+ cells increase in acute (A), subacute (B) and chronic (C) AA patients detected by Ki-67/tryptase double IHC.

Scale bars: 50µm.

Fold increase of MC density according to the histological features and clinical evaluation groups evaluated using Ki-67/tryptase IHC (D). Analysis derived from 81 areas (HFs) of 17 AA patients and 50 areas (HFs) of 7 healthy controls, ±SEM, One Way Anova (p<0.0001) (including the control values, black line) followed by Bonferroni´s Test (**p≤0.01, ***p≤0.001).

Fold increase of MC density according to the histological features and clinical evaluation groups evaluated using Ki-67/c-Kit IHC (E). Analysis derived from 31

areas of 30 HFs of 10 AA patients and 35 areas of 35 HFs of 7 healthy controls, ±SEM, One Way Anova ($p<0.0001$) (including the control values, black line) followed by Bonferroni´s Test (*$p\leq0.05$, **$p\leq0.01$, ***$p\leq0.001$). Black line indicates the control. The fold increase was calculated by dividing all the MC density values of acute, subacute, chronic AA and controls (n.of MC/mm2) with the mean value of the control CTS or PFD. AA patients were divided into three groups following their histological features [65] and clinical evaluations supplied by the dermatologist which are respectively acute (n=3-4), subacute (n=10) and chronic (n=3) stages.

Connective tissue sheath (CTS), hair follicle (HF), perifollicular dermis (PFD), sebaceous gland (SG).

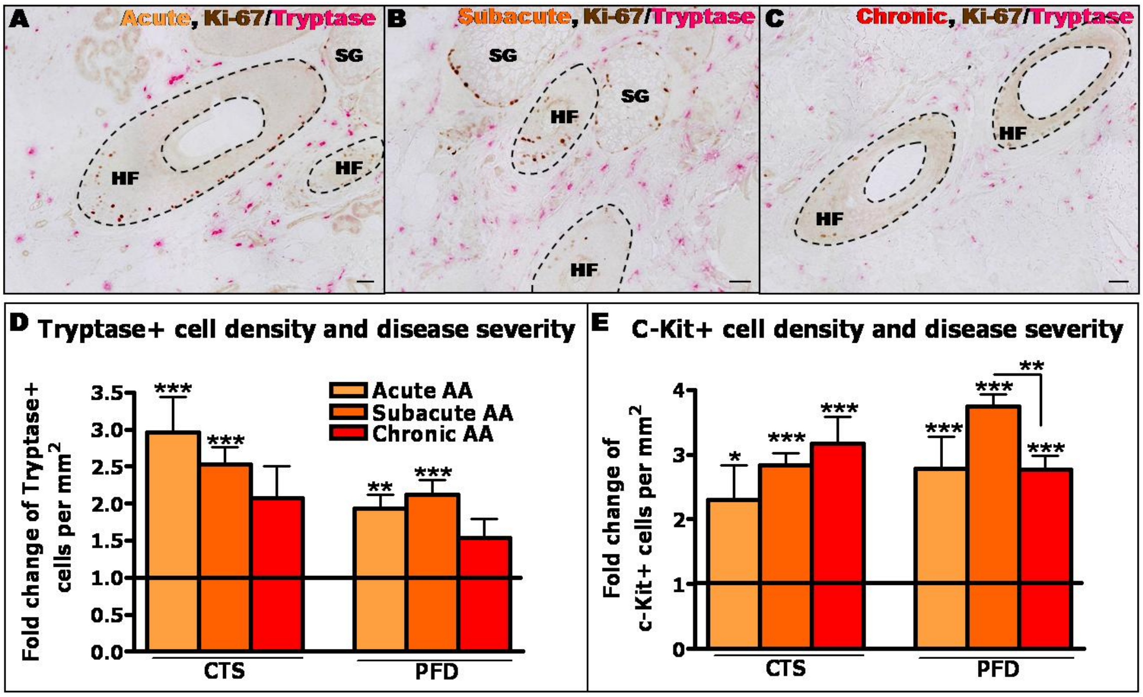

Supplementary Figure S4. TGFβ1 immunoreactivity is decreased in the HF ORS of AA patients.

Representative pictures of TGFβ1 IR in the HF ORS of healthy individuals (A) and AA patients (B). Scale bars: 100µm.

Quantitative (immuno-)histomorphometry by using Image J of TGFβ1 IR in AA HFs compared to healthy HFs (C).

Analysis derived from 41 areas of 21 HFs of 4 healthy controls and of 90 areas of 61 HFs of 17 AA patients and, ±SEM, Student t-Test, *p≤0.05, ***p≤0.001.

Connective tissue sheath (CTS), inner root sheath (IRS), outer root sheath (ORS).

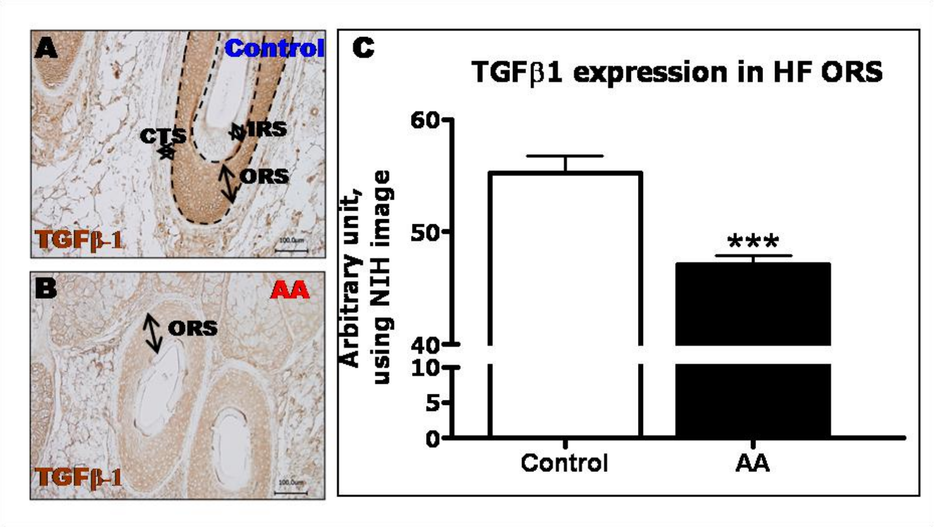

Supplementary Figure S5. MHCI/CD8/Tryptase triple staining showing MHC class I+ MCs in close contact with CD8+ T-cells

Representative pictures of MHCI/CD8/Tryptase in human tonsil (A-B), the positive control and AA skin (C-D). Higher magnification of MHC class I+ MCs in close contact with CD8+ T-cells in the small panels (C-D). Scale bars: 50µm.

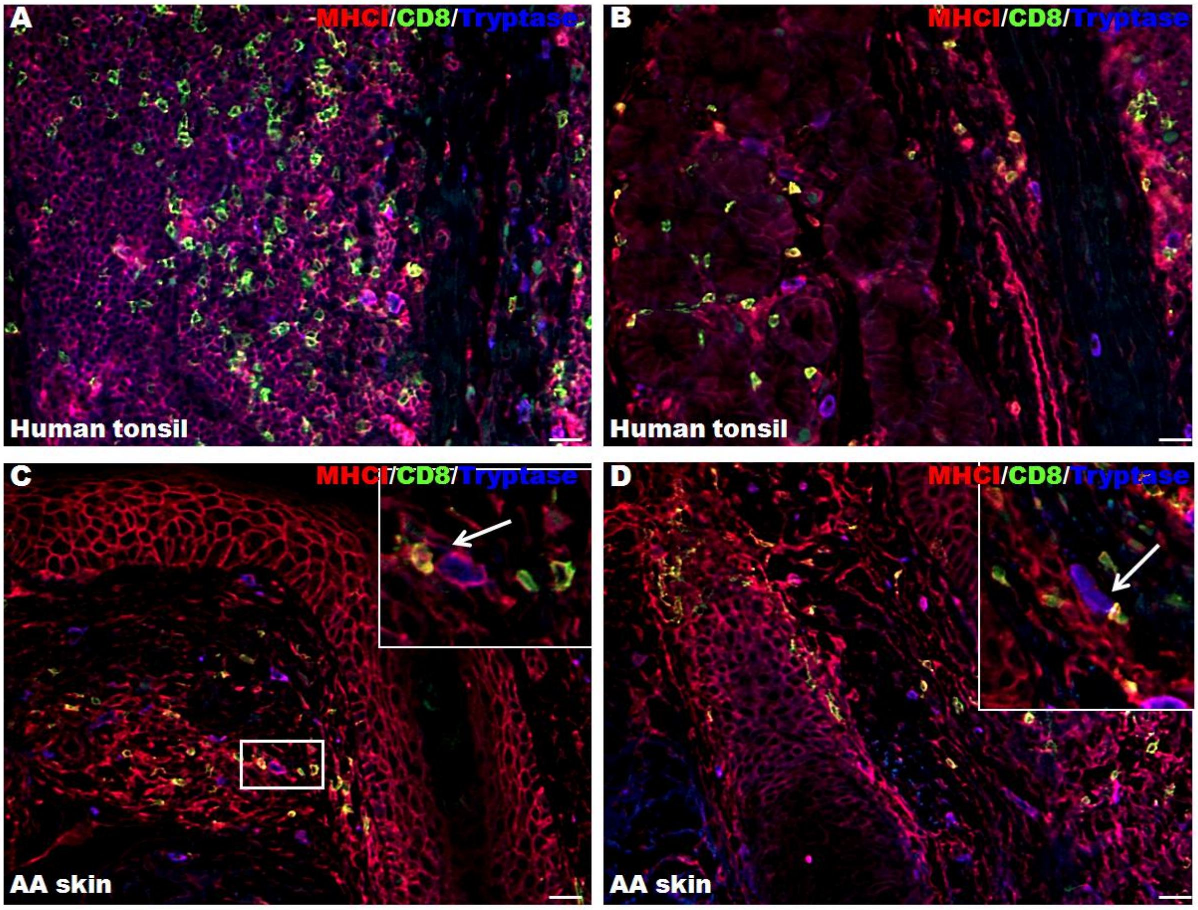
A
MHCI/CD8/Tryptase
Human tonsil
B
MHCI/CD8/Tryptase
Human tonsil
C
MHCI/CD8/Tryptase
AA skin
D
MHCI/CD8/Tryptase
AA skin

Supplementary Table S1. Primary Antibodies

Antibodies used for immmunohistochemical and immunofluorescence stainings are listed and described in detail.

Anti-human antibodies						
**Primary antibody**	**Origin**	**Clone**	**Vendor**	**Dilution**	**Sections**	**Reference**
**C-Kit**	Rabbit		DAKO	1:100<br>1:400	Paraffin	[47]
**Tryptase**	Mouse	AA1	Abcam, Cambridge, UK	1:500<br>1:1000	Paraffin	[47]
**Ki-67**	Mouse	Tec-3	DAKO	1:10	Paraffin	[60]
**TGFß1**	Rabbit		Santa Cruz	1:100	Paraffin	[58]
**MHCI**	Abcam	EMR8-5	Abcam	1:50	Paraffin	[169]
**CD8**	Mouse	C8/144B	DAKO	1:100<br>1:500	Paraffin,	[59]
**OX40L**	Mouse	159403	R&D Sytem	1:25	Paraffin	[76]
**CD30L**	Mouse	116614	R&D Sytem	1:50	Paraffin	[86]
**4-1BBL**	Rabbit		Abcam	1:200	Paraffin	[s35]
**ICAM-1**	Rabbit	EP1442Y	Abcam	1:100	Paraffin	[s40]
**IL-10**	Mouse	23738	R&D Sytem	1:25	Paraffin	[s37]
**PD-L1**	Mouse	29E2A3	BioLegend	1:100	Paraffin	[s41]
**CD200**	Goat		R&D System	1:200	Paraffin	[s42]
**Anti-mouse antibodies**						
**C-Kit**	Rat	2B8	BD Pharmigen	1:100	Cryo	[s43]
**CD8a**	Rat	53-6.7	BD Pharmigen	1:10	Cryo	[58]
**mMCP6**	Rabbit		M. Gurish	1:500	Cryo	[s44]

Supplementary Table S2.

Expression of pro-inflammatory and pro-inhibitory molecules and cytokines which are considered to be involved in the cross-talk between MCs and CD8+ T-cells in PFD of lesional AA skin compared to non-lesional AA and healthy skin.

Pro-inflammatory and immuno-inhibitory molecules which are considered to be involved in the cross-talk between MCs and CD8+ T-cells were analysed within MCs (IR inside MC). Moreover, positive MCs for these markers were counted around HFs (n. of + MCs) as well as their percentage among all MCs (% of + MCs). Finally, we investigated if MCs were positive for these markers during their interactions with CD8+ T-cells (n. of + MCs during CD8+ T-cells interactions) in AA patients. Arrows indicate increased (↑) or decreased (↓) expression and stars indicate significance (***p≤0.001, **p≤0.01, *p≤0.05), n.q. not quantified.

**Expression of pro-inflammatory and pro-inhibitory molecules in MCs and during their interactions with CD8+ T-cells in PFD of AA lesional skin compared to controls**				
**Antigens**	**IR inside MCs**	**Number (or %) of positive MCs**	**Number of MC positive for the indicated antigen during interaction with CD8+ T-cells**	**Selected background references**
**Pro-inflammatory**				
**Tryptase**	↑↑↑ (***)			[25]
**OX40L**		↑↑↑ (***)	↑↑ (*)	[80]
**4-1BBL**		↑(rare) (n.q)	↑(rare) (n.q)	[97]
**CD30L**		↑↑↑(***/*)	Almost never	[87]
**ICAM-1**		↑(few) (n.q)	Rare (n.q)	[99]
**Immuno-inhibitory**				
**TGFbeta1**	↓↓			[69]
**IL-10**	↓(n.q)	↓↓↓(***)	Almost never	[49]
**PD-L1**	↓(n.q)	↓ (n.q)	never	[106]
